# Divergent dynamics of inflammatory mediators and multiplex PCRs during airway infection in cystic fibrosis patients and healthy controls: Serial upper airway sampling by nasal lavage

**DOI:** 10.3389/fimmu.2022.947359

**Published:** 2022-11-18

**Authors:** Nina Erdmann, Theresa Schilling, Julia Hentschel, Thomas Lehmann, Philipp von Bismarck, Tobias Ankermann, Franziska Duckstein, Michael Baier, Carlos Zagoya, Jochen G. Mainz

**Affiliations:** ^1^ Cystic Fibrosis Centre, Brandenburg Medical School (MHB) University, Klinikum Westbrandenburg, Brandenburg an der Havel, Germany; ^2^ Jena University Hospital, CF-Center, Jena, Germany; ^3^ Institute of Human Genetics, Leipzig University Hospital, Leipzig, Germany; ^4^ Jena University Hospital, Center for Clinical Studies (Biometrics), Jena, Germany; ^5^ Klinik für Kinder- und Jugendmedizin I, Universitätsklinikum Schleswig-Holstein (UKSH), Kiel, Germany; ^6^ Jena University Hospital, Department of Medical Microbiology, Jena, Germany; ^7^ Faculty of Health Sciences, joint Faculty of the Brandenburg University of Technology Cottbus-Senftenberg, the Brandenburg Medical School Theodor Fontane and the University of Potsdam, Cottbus, Brandenburg an der Havel and Potsdam, Germany

**Keywords:** cystic fibrosis, interleukin, metalloproteinase, neutrophilic elastase, *Pseudomonas aeruginosa*, nasal lavage

## Abstract

**Background:**

In cystic fibrosis (CF), acute respiratory exacerbations critically enhance pulmonary destruction. Since these mainly occur outside regular appointments, they remain unexplored. We previously elaborated a protocol for home-based upper airway (UAW) sampling obtaining nasal-lavage fluid (NLF), which, in contrast to sputum, does not require immediate processing. The aim of this study was to compare UAW inflammation and pathogen colonization during stable phases and exacerbations in CF patients and healthy controls.

**Methods:**

Initially, we obtained NLF by rinsing 10 ml of isotonic saline/nostril during stable phases. During exacerbations, subjects regularly collected NLF at home. CF patients directly submitted one aliquot for microbiological cultures. The remaining samples were immediately frozen until transfer on ice to our clinic, where PCR analyses were performed and interleukin (IL)-1β/IL-6/IL-8, neutrophil elastase (NE), matrix metalloproteinase (MMP)-9, and tissue inhibitor of metalloproteinase (TIMP)-1 were assessed.

**Results:**

Altogether, 49 CF patients and 38 healthy controls (HCs) completed the study, and 214 NLF samples were analyzed. Of the 49 CF patients, 20 were at least intermittently colonized with *P. aeruginosa* and received azithromycin and/or inhaled antibiotics as standard therapy. At baseline, IL-6 and IL-8 tended to be elevated in CF compared to controls. During infection, inflammatory mediators increased in both cohorts, reaching significance only for IL-6 in controls (p=0.047). Inflammatory responses tended to be higher in controls [1.6-fold (NE) to 4.4-fold (MMP-9)], while in CF, mediators increased only moderately [1.2-1.5-fold (IL-6/IL-8/NE/TIMP-1/MMP-9)]. Patients receiving inhalative antibiotics or azithromycin (n=20 and n=15, respectively) revealed lower levels of IL-1β/IL-6/IL-8 and NE during exacerbation compared to CF patients not receiving those antibiotics. In addition, CF patients receiving azithromycin showed MMP-9 levels significantly lower than CF patients not receiving azithromycin at stable phase and exacerbation. Altogether, rhinoviruses were the most frequently detected virus, detected at least once in n=24 (49.0%) of the 49 included pwCF and in n=26 (68.4%) of the 38 healthy controls over the 13-month duration of the study. Remarkably, during exacerbation, rhinovirus detection rates were significantly higher in the HC group compared to those in CF patients (65.8% vs. 22.4%; p<0.0001).

**Conclusion:**

Non-invasive and partially home-based UAW sampling opens new windows for the assessment of inflammation and pathogen colonization in the unified airway system.

## 1 Introduction

In cystic fibrosis (CF), the absence or functional deficiency of the cystic fibrosis transmembrane conductance regulator (CFTR) protein (1) leads to dysfunctional electrolyte and fluid excretion from cells, resulting in viscous body fluids (2, 3). Beside abdominal and gonadal exocrine glands, these viscous secretions affect the airway system, which, by pulmonary destruction, is responsible for premature death in 90% of patients (4, 5). The resulting impaired mucociliary clearance (6) facilitates colonization with pathogens in upper (UAW) and lower airways (LAW) of people with CF (pwCF) (3, 5, 7). Resulting symptoms in the UAW are chronic nasal congestion, rhinorrhea with anterior and postnasal drip, mouth breathing, and anosmia, which deteriorate life quality and overall health (8, 9). On a biochemical level, impaired mucociliary clearance and chronic infections driven by pathogens lead to neutrophilic inflammation and progressive bronchiectasis, still the main cause of premature death in pwCF (3, 6). During the preceding years, we gained extensive knowledge about the pathomechanisms of UAW and LAW inflammation: while chronic infections in LAW mainly lead to polymorphonuclear neutrophil (PMN)-triggered response (10, 11), resulting in the release of huge amounts of proteases, i.e. neutrophil elastase (NE) (12) and matrix metalloproteinase 9 (MMP-9) (12, 13) and building neutrophil extracellular traps (NETs) (14), UAW defense mechanisms seem to act differently. Here, the inflammatory response appears to be mainly immunoglobulin A (IgA)-triggered, whereas PMNs only play a secondary role (14). Furthermore, recent studies reveal UAWs as an important entry point and reservoir for pathogens like *Pseudomonas aeruginosa* and *Staphylococcus aureus*, where pathogens can adapt and from where re-infection can occur even after lung transplantation (unified airways hypothesis) (15, 16). This is important because chronic bacterial airway infections with pathogens like *P. aeruginosa* and *S. aureus* aggravate inflammation and lead to pulmonary endothelial changes resulting in the deterioration of lung function and pulmonary destruction (17).

Following current guidelines (18, 19), pwCF chronically colonized with *P. aeruginosa* are aggressively treated with daily inhaled antibiotics (tobramycin, colistin, or aztreonam lysine). Although this treatment cannot eradicate the pathogen, it reduces bacterial counts, thereby delaying pulmonary inflammation and destruction and therefore improving life expectancy. Furthermore, long-term treatment with oral azithromycin is a standard in CF therapy for chronically colonized patients (20). Although *P. aeruginosa* is resistant to this macrolide, its long-term therapy has been shown to reduce the overwhelming inflammatory immune response in the host (21).

Concepts on how to manage airway colonization with *S. aureus* in pwCF are more heterogenous among CF centers. Some programs are less aggressive regarding *S. aureus*, although some studies have revealed increased (UAW) inflammation (22). This is because lung function does not decrease to a similar extent as in *P. aeruginosa* colonization (17) and the pathogen is mostly sensitive to many antibiotics that can be orally administered.

In addition to the above-mentioned pathogens, there is increasing evidence that respiratory viral infections play a crucial role as cause of acute exacerbations in CF than formerly assumed (23). They have been associated with disease progression, hospitalization, longer courses of intravenous antibiotic therapy, and, finally, with impaired pulmonary function and reduced life expectancy in the inherited disease (24–27). Furthermore, although viral infections may occur independently from bacterial infections, they are suspected to be a risk factor for bacterial colonization and infection with pathogens like *S. aureus* (24) or *P. aeruginosa* (28–30). Finally, in pwCF with chronic airway colonization with these pathogens, viral infections are known to enhance bacterium-driven exacerbations and, thereby, pulmonary destruction as the primary reason for premature death in pwCF (31–33).

The objective of this prospective study was to assess and compare inflammatory dynamics in the airway system assessed by nasal lavage fluid (NLF) of CF patients and healthy controls during stable phase and exacerbation/acute respiratory infection (ARI) with regard to different factors such as bacterial colonization in pwCF and viral infections in both cohorts. Furthermore, the relation between inflammation and chronic antibiotic therapies, like inhaled and azithromycin therapies, is also assessed. NLF has already been implemented in UAW monitoring in chronic lung diseases like chronic obstructive pulmonary disease (COPD) and bronchial asthma, and it is being further established also in CF. As a non-invasive, inexpensive, and readily accessible method, it is particularly useful for monitoring children (20, 34).

## 2 Materials and methods

### 2.1 Patients

CF patients and healthy controls were prospectively included between 01/2013 and 02/2014. The recruitment of pwCF took place during regular outpatient visits to the Jena University CF Center. Healthy controls were recruited by a student from the CF center and other staff of the Jena University Hospital. CF diagnosis of patients relied on two positive sweat tests and/or identification of two disease-causing mutations in the *CFTR* gene locus.

Acute pulmonary exacerbation was defined according to Fuchs criteria in its modified version (35). A patient was considered to have an exacerbation when 4/12 of the following signs or symptoms were met: change in sputum, hemoptysis, increased cough, increased dyspnea, malaise/fatigue or lethargy, body temperature >38°C, weight loss/loss of appetite, sinus pain, altered sino-nasal secretions, change in physical examination of the chest, and decrease of 10% or more in forced expiratory volume in 1 s (FEV_1_) compared to previous test results and radiographic signs indicating pulmonary infection.

Inclusion criterion for stable/healthy phase was the absence of symptoms for at least 14 days prior to nasal lavage collection. Exclusion criterion for the control cohort was the diagnosis of CF, primary ciliary dyskinesia, or immunodeficiency. Subjects with tympanic membrane perforation were excluded from both cohorts. Treatment with inhaled or oral antibiotics, including azithromycin, was ascertained but not regarded as exclusion criterion. In contrast, current administration of intravenous antibiotics was an exclusion criterion for “stable phases.” Further medical treatment was documented including nasally or pulmonary applied topical steroids.

All subjects below the age of 18 years were included in the pediatric cohort and subjects aged at least 18 years in the adult cohort. For evaluation, subjects turning 18 years old during the study remained in the pediatric cohort.

Written informed consent was obtained from each patient or his/her legal guardian, and the study was approved by the local ethics committee (technical opinion number 3608-11/12) in accordance with the 1964 Helsinki Declaration. The study was registered on Clinical trials.gov (NCT00803881).

### 2.2 Nasal lavage collection

**Figure 1:**
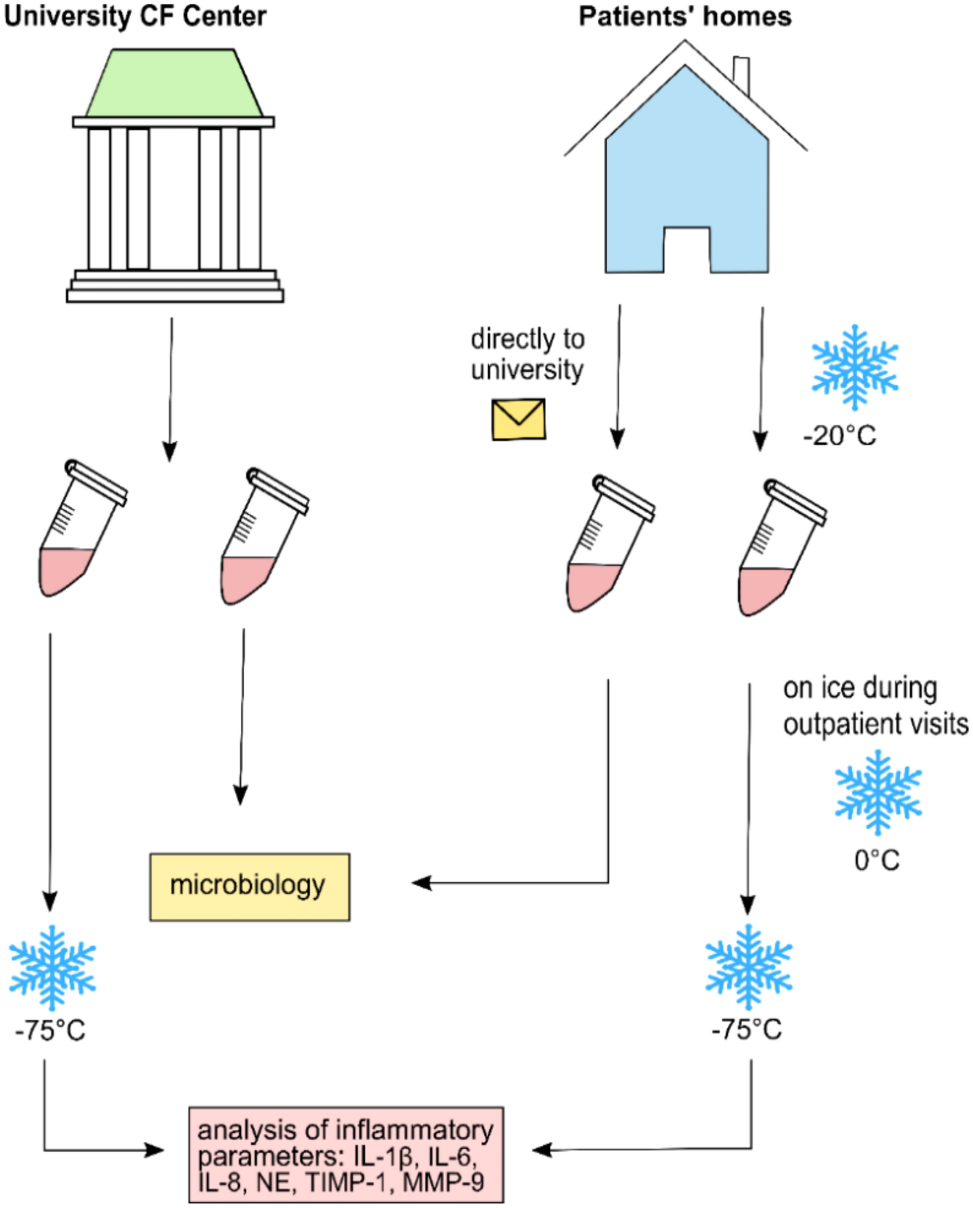
Collection and transference procedure for nasal lavage fluid samples analyzed in this study.

NLF was obtained according to the procedure described in previous publications (36). In brief, using a syringe, 10 ml of sterile isotonic saline was applied to each nostril. Patients were told to hold their head in a slightly backward position for 10 s. During the procedure, the soft palate was temporarily closed forming a “K”-sound, preventing fluid from dripping into the oropharynx. Thereafter, while leaning slightly forward during exhalation, NLF was rinsed into a sterile beaker. If conventional nasal lavage (NL) was not possible because of age and/or compliance, we applied compressor-assisted NL using a large drop-nebulizing device with 10 ml isotonic saline (Rhinoclear^®^). NLF was collected, and samples were treated as mentioned above. Samples were either taken during regular outpatient clinic visits where most aliquots were directly frozen at −75°C or at home by the patients/families. When samples were taken from CF patients at home, one aliquot was separated and directly sent to the laboratory for conventional microbiological analysis. The remaining samples were immediately frozen at home at −20°C and later transferred on ice to our clinic for assessment of inflammatory mediators. Further storage for all samples occurred at −75°C until analysis (**Figure 1**).

### 2.3 Analyses of microbiological cultures

Bacterial pathogen detection in pwCF was performed *via* conventional microbiology using native NLF samples in the Jena University Hospital Department of Microbiology, following the current standards. The extent of bacterial colonization, i.e. intermittent or chronic, was defined according to Lee criteria (37): chronic infection was defined as >50% of positive microbiological examinations during the preceding 12 months and intermittent infection as <50% of positive microbiological examinations during the preceding 12 months (at least three samples taken/year). However, both groups were later combined due to their small sample size. Modified Lee criteria were also applied on every bacterium found in the UAW. As only 1/49 pwCF was found positive with methicillin-resistant *S. aureus* (MRSA), no distinction was made between colonization with MRSA and methicillin-sensitive *S. aureus* (MSSA). Similarly, UAW colonizations of non-mucoid and mucoid *P. aerugino*sa were combined. During this study, *P. aeruginosa* colonization was detected in 16/49 patients. Additionally, four patients carried *P. aeruginosa* intermittently during the preceding year, according to retrospective chart review.

The physiological flora of UAW was defined according to (38, 39). Bacteria causing exacerbation and ARI were defined according to Goss et al., Zemanick et al., and others (40–42). For microbiological analysis, specimens were processed at the Jena University Hospital microbiology laboratory according to the German quality assurance guidelines for CF microbiology (43, 44).

### 2.4 Analysis of bacterial and viral pathogens *via* PCR

Nineteen bacterial and viral pathogens known to cause acute airway infections/exacerbations were detected using a real-time multiplex PCR (m-RT-PCR) instrument (Roche, Mannheim, Germany) at the University Hospital of Schleswig-Holstein, Kiel, Germany. Viral pathogens targeted by PCR were adenovirus, enterovirus, influenza virus A (including H1N1) and B, parainfluenza viruses 1–4, respiratory syncytial virus (RSV), human rhinovirus (hRV), metapneumovirus (hMPV), coronaviruses 229E and OC43, and human bocavirus. Bacterial pathogens targeted by PCR were *Chlamydia pneumoniae*, *Legionella pneumophila*, *Bordetella pertussis*, *Bordetella parapertussis*, and *Mycoplasma pneumoniae* (45).

In six samples (one stable phase sample and five exacerbation samples, respectively) in which conventional microbiology could not be performed due to an insufficient amount of material, multiplex PCR analysis of viral- and pneumonia-causing pathogens was conducted using the Unyvero Hospitalized Pneumonia (HPN) Panel (Curetis GmbH, Holzgerlingen, Germany). Samples were thawed at room temperature and treated according to manufacturer guidelines.

### 2.5 Cytokine analysis

NLFs were thawed and centrifuged at 1,000 rpm for 10 min. Cytokine levels were measured *via* bead-based multiplex assay using the Milliplex MAP-Kits^®^ (Human High Sensitivity HSTC-MAG-28K, Human MMP Panel 2 HMMP2MAG-55K, Human TIMP Panel 1 HTMP1MAG-54 K, Merck Millipore, Darmstadt, Germany). Fluorescence analysis was conducted using a fluorimeter (Bio-Plex^®^200 System, Bio-Rad, CA, USA). Concentrations of IL-1β, IL-6, IL-8, matrix metalloproteinase (MMP)-9, and tissue inhibitor of metalloproteinase (TIMP)-1 were determined according to instructions provided by the manufacturer. As specified by the manufacturer, minimum detection limits were 0.12 pg/ml (IL-1β), 0.13 pg/ml (IL-6), 0.12 pg/ml (IL-8), 2.0 pg/ml (MMP-9), and 4.0 pg/ml (TIMP-1). Samples below the minimum detection limit were replaced by their respective detection limit minus 0.1 pg/ml. Samples above 1.5 times of the maximum detection limit were diluted (dilution factors, 1:5 to 1:10) with isotonic (for IL-1ß, IL-6, and IL-8) or buffered saline (for MMP-9 and TIMP-1) before rerun. In 21/214 samples, further dilution did not lead to decreased values. In these cases, the maximum value in the series of measurements of the respective parameter plus 0.1 pg/ml was assumed. Neutrophil elastase (NE) was determined using Human PMN Elastase ELISA (DEH3311, Demeditec Diagnostics GmbH, Kiel, Germany). The detection limit specified by the manufacturer was 0.2 ng/ml. All samples were used undiluted, and measurements were taken out in duplicates. Spectroscopy analyses were performed using FLUOstar Galaxy Spectrometer (BMG LABTECH GmbH, Offenburg, Germany).

### 2.6 Statistical analysis

Statistical analyses were performed with SPSS 27 (IBM, Ehningen, Germany), MS Excel (Redmont, USA), and GraphPad Prism 8 (La Jolla, USA). Sample sizes for the patient and healthy control cohorts were calculated with preliminary data of IL-1β, IL-6, IL-8, NE, TIMP-1, and MMP-9 in NL of 12 patients and 7 healthy controls at exacerbation. Given that such data were not normally distributed, sample size calculations were based on medians of such inflammatory parameters, according to (46) and assuming standard deviations equal to 0.5-fold of the median of each respective inflammatory parameter. Of all inflammatory parameters, NE yielded the largest estimated sample size (n=35) to attain a minimum power level of 0.8. Therefore, considering a dropout rate of approximately 10%, i.e., approximately n=3, we arrived at an estimated minimal sample size of n=38. Descriptive statistics of demographic data was reported as means and their standard deviations. Comparisons between percentages of individuals infected with bacterial, fungal, and viral pathogens were conducted using Pearson chi-squared tests. Results from comparisons of inflammatory markers are reported as medians and interquartile ranges (IQRs). Statistical significance was determined using mixed analysis of variance (ANOVA) on ranks for comparisons of inflammatory markers in CF patients and healthy controls at stable and exacerbation phases, and children and adult subgroups. When the normality assumption for mixed ANOVA was not met (as in IL-1β and IL-8 cases), log10 transformations were used. Statistical analyses of inflammatory markers between CF subgroups (CF patients receiving and not receiving antibiotics, with *P. aeruginosa* colonization, and with viral infections) were performed *via* Mann–Whitney U-tests. Odds ratio calculations were performed using the generalized estimating equations method for repeated measures and assuming an unstructured correlation matrix. p-values <0.05 were considered statistically significant.

## 3 Results

### 3.1 Study population

During the study period, a total of 214 NLF samples, obtained from 49 pwCF (126 NLF samples) and 38 healthy controls (88 NLF samples), were collected at five time points (see **Supplementary Table S7**) with a mean of 2.5 NL per patient in the CF cohort (range, 2–5) and a mean of 2.2 NL in controls (range, 2–5). Sex ratio in both groups was non-significantly imbalanced in favor of female individuals in the CF cohort (22m, 27f). This imbalance was even more pronounced in controls (12m, 26f). Mean age at study entry was similar in both cohorts.

Among pwCF, 21/49 (43%) were homozygous and 26/49 (53%) were heterozygous for the most common mutation F508del. A total of 2/49 (4%) patients had rare non-F508del *CFTR* mutations (R347P/2183AA->G and G542X/D110H). Twenty-seven of 49 (55%) pwCF suffered from chronic rhinosinusitis (CRS) according to the European Position Paper on Rhinosinusitis and Nasal Polyps (EPOS) criteria including nasal blockage and discharge, postnasal drip, fascial pain, headache, and loss/reduction in sense of smell (47). Lung function testing during the study period resulted in a mean FEV_1_ of 79.6 ± 30.9% (range, 21.5%–125.7%) during stable phases and 72.3 ± 30.2% (range, 22.0%–116.8%) during exacerbations. Twenty of 49 (40.8%) pwCF were intermittently or permanently colonized with *P. aeruginosa*, 16/49 (32.7%) were tested positive during trial, and 12/20 (60%) were adults. As a specific standard of the CF center, patients who tested positive for *P. aeruginosa* received pulmonary applied antibiotics for 1 year, following the last positive culture. Thirty-five of the 49 (71%) patients had a known *S. aureus* colonization; 28/35 (80%) of those were children. Ten of the 49 (20%) patients were co-infected in upper and lower airways with both pathogens. Patients colonized with *P. aeruginosa* received oral azithromycin and/or inhaled colistin, tobramycin, or aztreonam lysine as a standard therapy, according to the current standards of care (18). None of the patients received CFTR modulating therapy, as the study was completed before approval in Germany. Demographic data of the included patients are shown in **Table 1** and in **Supplementary Tables S1, S2**.

**Table 1:** Demographic data of included CF patients and healthy controls.

Nominal variables	CF patients		Healthy controls		p
**Age at inclusion** (mean ± SD)	19.0 ± 14.8 years		22.7 ± 16.3 years		>0.05
**Pediatric subgroup** (<18 years)	n=30 (61.2%)		n=14 (36.8%)		**<0.05**
Mean age ± SD:	9.6 ± 4.4 years		5.2 ± 4.5 years		**<0.01**
Range:	3 – 17 years		1 – 17 years		
**Adults** (18 years and older)	n=19 (38.8%)		n=24 (63.2%)		<0.05
Mean age ± SD:	34.9 ± 12.3 years		33.3 ± 10.9 years		>0.05
Range:	22 – 75 years		22 – 58 years		
**Gender**					
Male	22/49	44.9%	12/38	31.6%	>0.05
Female	27/49	55.1%	26/38	68.4%	>0.05
**CFTR genotype**					
Delta F508/delta F508	21/49	22.9%			
Delta F508/others	26/49	53.0%			
Other/other	2/49	4.0%			
**Steroids**					
Oral (systemic)	4/49	8.2%			
Pulmonal (topic)	22/49	44.9%			
Nasal (topic)	29/49	59.2%			
**Medical record**					
Chronic rhinosinusitis (CRS)	27/49	55.1%	2/38	5.2%	**<0.01**
Allergy	12/49	24.5%	11/38	29.0%	>0.05
ABPA	8/49	16.3%	0/38	0.0%	n.a.^†^
Asthma bronchiale	1/49	5.2%	2/38	5.2%	>0.05
Diabetes mellitus	13/49	26.5%	0/38	0.0%	n.a.^†^
History of lung transplantation	1/49	5.2%	0/38	0.0%	n.a^†^

ABPA, allergic bronchopulmonary aspergillosis; SD, standard deviation.

^†^No p-values were computed for these cases, as frequencies in healthy cohort were null.

Bold values stand for statistically significant result.

n.a, not applicable.

### 3.2 Frequency of airway exacerbation/infection during the trial

During the 13-month trial, we found a mean of 1.5 ± 0.6 exacerbations in the CF cohort and 1.4 ± 0.8 ARIs in the control cohort. There was no significant difference between both cohorts, but as expected, children revealed to have slightly higher infection rates than adults.

### 3.3 PCR detection of viral and bacterial pathogens during stable phases and exacerbation

PCR analyses were performed in 213 of the 214 NLF samples collected over the 13-month study. During that period, out of the 14 viruses targeted by PCR, 5 were detected in the CF cohort (hRV, adenovirus, enterovirus, parainfluenza virus type 2, and hMPV), and 4 four were detected in the HC cohort (hRV, adenovirus, hMPV, and coronavirus). Among the n=31 (63.3%) pwCF who tested positive for any virus at least once during the whole observation period, n=23 (46.9%) tested positive for a single virus, n=6 (12.2%) for two different viruses, and n=2 (4.1%) for three different viruses. Over the whole study, 18 (36.7%) pwCF remained negative for all the targeted viral pathogens. Regarding the HC cohort, n=19 (50%) out of the 38 considered subjects tested positive for a single virus and n=9 (23.7%) for two different viruses. There were no subjects in the HC cohort with detection of more than two different viruses during the whole observation time frame, and n=10 (26%) individuals remained negative for all the viruses targeted in this study. Individuals infected with a viral pathogen at least once during the whole study were significantly younger compared to subjects who remained free of such pathogens (median, IQR: 14.3 [5.8, 27.3] years vs. 24.5 [15.7, 37.4] years; p=0.02).

The most frequently detected viral pathogen over the whole study was hRV in both groups, being detected at least once in n=24 (49.0%) of the 49 included pwCF and in n=26 (68.4%) of the 38 healthy individuals included in the study. In general, median age in HC subjects (median, IQR: 23.4 [4.4, 32.3] years) who became infected with hRV at least once during the whole study was greater than in pwCF (median, IQR: 11.3 [8.6, 25.0] years), but such a difference was not statistically significant.

At stable phases, hRV detection rates were equally distributed in both groups (18.4%). In contrast, during the first exacerbation, hRV detection rates were significantly higher in the HC group than in the CF group (65.8% vs. 22.4%; p<0.0001). Odds ratio calculations revealed that, at exacerbation, the probability for hRV to be detected in the healthy cohort was 6.6-fold higher than in the CF cohort [Wald confidence interval: (2.6, 17.2); p=0.005]. At stable phases and during exacerbation, the median age in the subgroup of individuals who tested positive for hRV was significantly lower than that in hRV-negative individuals (stable phase median, IQR: 8.9 [4.5, 21.1] years vs. 23.1 [9.7, 31.8] years, p=0.043; exacerbation median, IQR: 13.8 [4.7, 26.4] years vs. 23.1 [10.2, 35.3] years, p=0.018). At subsequent exacerbation time points, differences in hRV detection rates and in infection ages did not attain significance due to the small number of collected samples at those time points (**Supplementary Table S8**).

Altogether, adenoviruses and enteroviruses were detected less frequently (**Supplementary Table S8**). Furthermore, analyses of hRV detection with regard to azithromycin intake revealed that pwCF not receiving azithromycin (n=34) had a much higher probability for hRV detection compared to pwCF receiving azithromycin (n=15) (odds ratio, 5.1; p=0.033).

Regarding bacterial pathogens targeted by PCR, only one subject in the CF cohort tested positive for *M. pneumonia* during exacerbation, whereas during stable phases, no subject tested positive for those pathogens.

### 3.4 Inflammatory parameters in NL

Given that not all participants were able to provide further NLF samples at the second and subsequent exacerbation time points, inflammatory markers analyses were restricted only to the first exacerbation time point, which is referred to only as exacerbation throughout the next sections in the paper.

#### 3.4.1 Inflammation markers in CF and healthy controls during stable phases and during exacerbation

**Figure 2:**
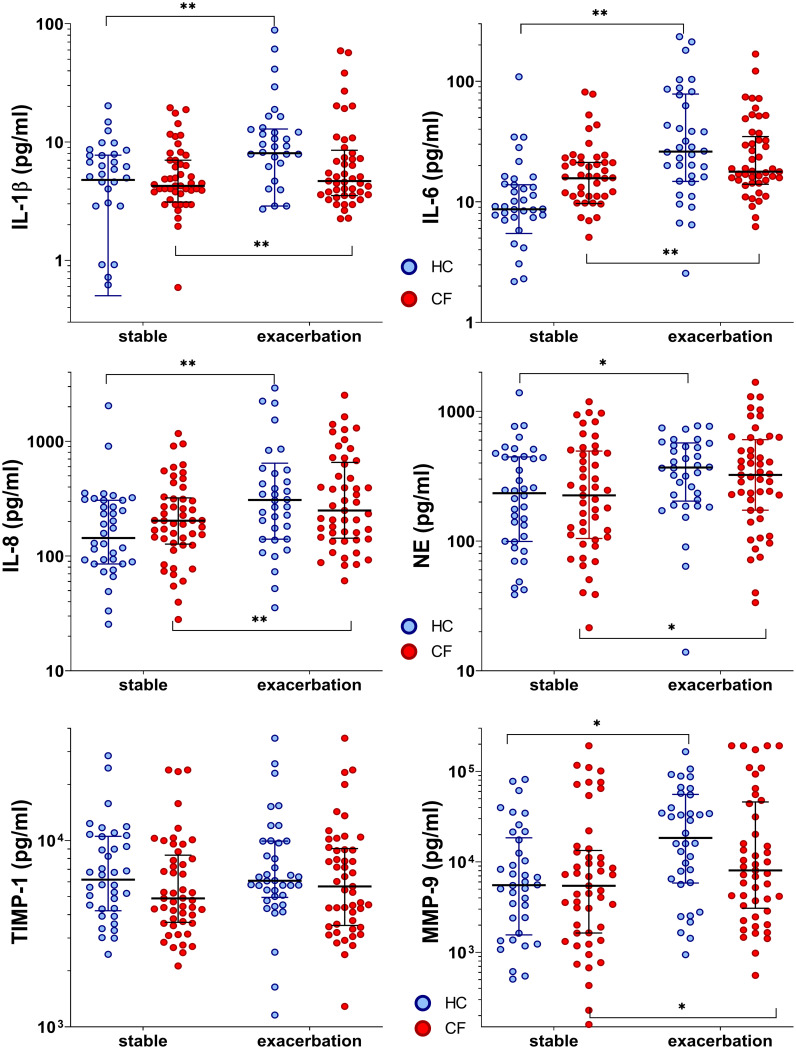
Inflammatory markers in healthy controls and people with CF during stable phase and exacerbation. Statistical significance levels are displayed as follows: ^*^p <0.05, ^**^p<0.01.

In the CF cohort, the baseline pro-inflammatory cytokines (IL-6 and IL-8) values tended to be elevated during stable phases compared to controls. IL-1β, IL-6, IL-8, NE, and MMP-9 levels in both cohorts increased significantly when entering exacerbation phases (median increase rate, 1.1 (IL-1β) to 4.4 (MMP-9); all p<0.05) (**Supplementary Table S3**). Furthermore, a significant interaction between the phase and cohort was found for IL-6 (p=0.047), revealing a more pronounced effect in the control group. During infections, mediators increased in both cohorts. Thereby, controls tended to reveal higher inflammatory responses during ARI with a broader range (increase rate, 1.6 (NE) to 4.4 (MMP-9), only TIMP-1 remained without increase). In the CF cohort, mediators increased only slightly by 1.2–1.5 times (IL-6, IL-8, NE, TIMP-1, and MMP-9), attributable to their elevated baseline levels (**Figure 2** and **Supplementary Table S3**).

#### 3.4.2 Inflammation markers in children and adults in CF and healthy controls during stable phase and exacerbation/ARI

Altogether, children showed elevated levels of pro-inflammatory cytokines in stable phases and during infections compared to adults. However, when entering exacerbation phases, IL-8 levels increased significantly in the CF children subgroup (n=30) and in child (n=14) and adult (n=24) control subgroups (median increase rate, 1.5 (children) to 2.5 (adults); p<0.001). Furthermore, MMP-9 was observed to increase significantly in child and adult control cohorts reaching exacerbation phases (median increase rate, 2.1 and 3.8, respectively; p<0.001). In contrast, significantly increased IL-1β values were observed only in children entering exacerbation phases (median increase rate, 2.1; p=0.001). On the other hand, significantly increased IL-6 values were detected only in healthy adults (median increase rate, 3.8; p<0.001) (**Supplementary Table S4**).

### 3.5 Virus detection and soluble inflammatory parameters

#### 3.5.1 Stable phase

When considering both CF and HC cohorts as a whole (n=87) during stable phases, the subjects who tested positive (n=24) for any of the six detected viruses were observed to have significantly higher levels of IL-6 (median, IQR: 14.5 (8.0, 31.3) pg/ml vs. 11.1 (7.2, 16.3) pg/ml; p=0.048). IL-1β, IL-8, NE, TIMP-1, and MMP-9 also tended to be elevated, but these results did not reach statistical significance. Furthermore, subjects who tested positive for hRV (n=16) showed significantly higher levels of IL-6 (median, IQR: 22.2 (9.5, 34.4) pg/ml vs. 11.1 (7.4, 16.3) pg/ml; p=0.02) and IL-8 (median, IQR: 290.7 (157.9, 345.3) pg/ml vs. 171.7 (87.2, 267.9) pg/ml; p=0.01). In contrast, the detection of any other virus different from hRV (n=11) was not associated with differences in any of the analyzed inflammatory parameters.

**Figure 3:**
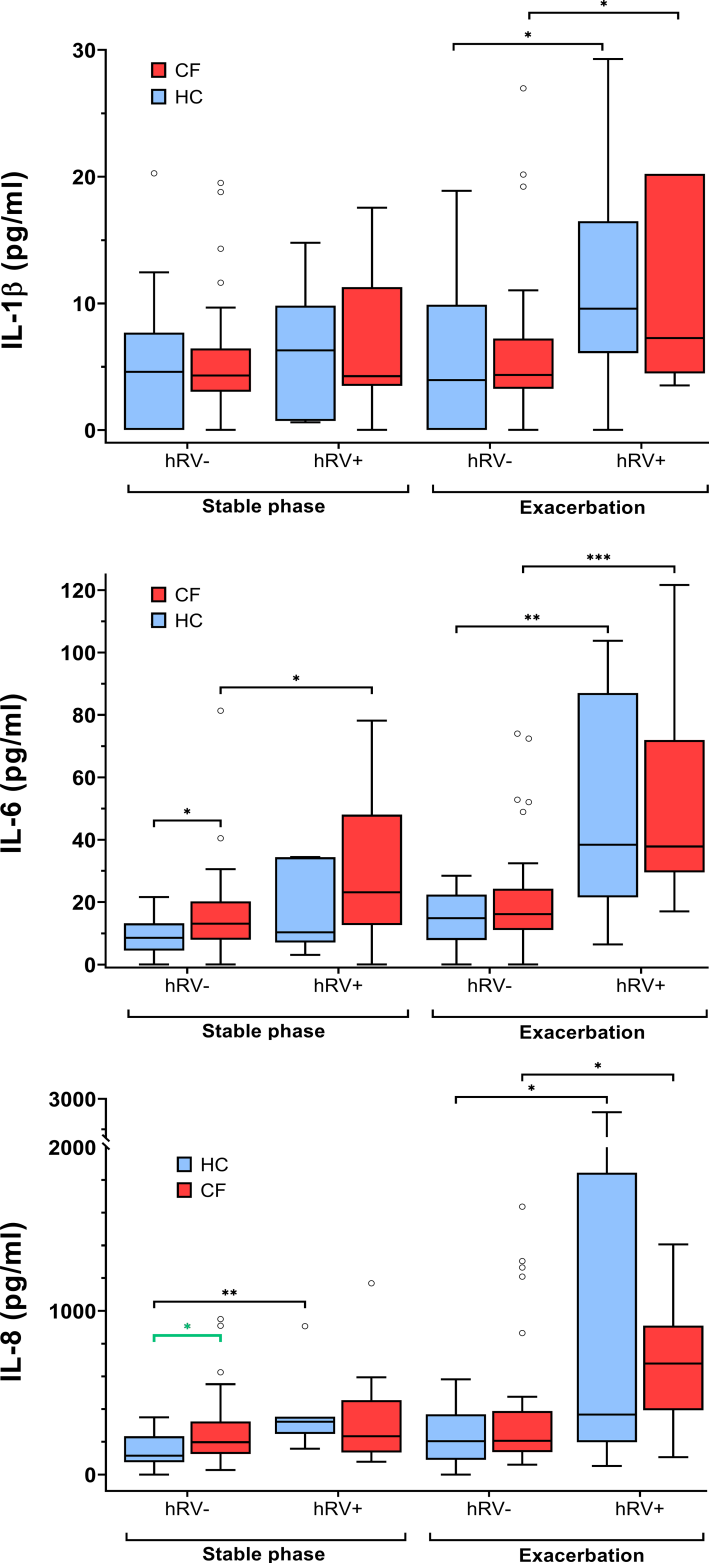
Inflammatory markers in pwCF and HC with regard to hRV detection. Statistical significance levels are displayed as follows: ^*^p <0.05, ^**^p<0.01, ^***^p<0.001.

Levels of IL-6 in the subgroup of pwCF with hRV (n=9) were significantly higher compared to pwCF without hRV detection. This contrasted with the inflammatory responses observed in the subgroup of healthy controls who tested positive for hRV infection (n=7) and showed significantly elevated levels of IL-8 and MMP-9 (**Figure 3** and **Supplementary Table S9**).

Furthermore, IL6 and IL-8 were significantly higher (p=0.02 and p=0.03, respectively) in the hRV-negative subgroup of pwCF than in the hRV-negative subgroup of HC. In contrast, at stable phases, MMP-9 concentrations were higher in the hRV-positive subgroup of HC than in the hRV-positive subgroup of pwCF (p=0.02) (**Figure 3** and **Supplementary Table S9**).

#### 3.5.2 Exacerbation

When considering the cohort consisting of both pwCF and HC as a whole at exacerbation, the subgroup who tested positive (n=43) for any of the above-mentioned viruses revealed significantly higher levels of IL-1β (median, IQR: 8.2 (4.6, 13.0) pg/ml vs. 4.1 (3.0, 7.0) pg/ml; p=0.01), IL-6 (median, IQR: 32.5 (19.4, 74.5) vs. 15.8 (10.4, 22.4) pg/ml; p<0.00001), IL-8 (median, IQR: 394.0 (216.4, 859.7) pg/ml vs. 205.0 (123.6, 362.6) pg/ml; p=0.001), NE (median, IQR: 449.2 (272.4, 633.6) pg/ml vs. 263.5 (160.1, 422.8) pg/ml; p=0.003), and MMP-9 (median, IQR: 31,529.6 (8,580.1, 65,830.2) pg/ml vs. 6,688.6 (2,501.2, 15,930.6) pg/ml; p=0.001). These differences again were mainly driven by individuals infected with hRV, as the detection of any virus different from rhinovirus (n=11) was not associated with differences in any of the analyzed inflammatory parameters. In contrast, rhinovirus detection (n=36) was significantly associated with higher levels of IL-1β (median, IQR: 9.1 (4.7, 16.5) pg/ml vs. 4.2 (3.0, 7.3); p=0.0004), IL-6 (median, IQR: 38.3 (24.4, 84.0) pg/ml vs. 16.1 (11.0, 21.8); p<0.00001), IL-8 (median, IQR: 469.9 (223.7, 971.7) pg/ml vs. 206.6 (134.5, 384.4) pg/ml; p=0.001), NE (median, IQR: 416.0 (272.4, 640.0) pg/ml vs. 298.4 (172.0, 465.0) pg/ml; p=0.01), and MMP-9 (median, IQR: 32,349.5 (8,580.1, 90,049.7) pg/ml vs. 7,208.8 (2,659.2, 18,067.2) pg/ml; p=0.002).

The subgroup of pwCF who tested positive for a viral infection (n=18) revealed higher inflammatory responses in IL-6 (median, IQR: 37.8 (29.9, 66.0) pg/ml vs. 16.1 (11.1, 23.5) pg/ml; p=0.001), IL-8 (median, IQR: 678.6 (444.7, 815.9) pg/ml vs. 206.7 (138.2, 386.3) pg/ml; p=0.028), NE (median, IQR: 636.5 (263.8, 834.2) pg/ml vs. 305.2 (148.7, 438.4) pg/ml; p=0.01), and MMP-9 (median, IQR: 47,804.5 (10,073.2, 151,285.5) pg/ml; p=0.01) compared to the subgroup of pwCF who tested negative for any of the mentioned viruses (n=31).

On the other hand, hRV infection in pwCF was associated with higher levels of IL-1β, IL-6, IL-8, NE, and MMP-9 (**Figure 3** and **Supplementary Table S9**), whereas the detection of any other virus different from hRV was not associated with differences in inflammatory responses.

The detection of rhinovirus (n=25) in the HC was associated with higher levels of IL-1β, IL-6, and IL-8. In the HC subgroup with hRV detection (**Figure 3** and **Supplementary Table S9**), one individual was concomitantly infected with an adenovirus and another one with hMPV.

### 3.6 Bacterial cultures and soluble inflammatory markers in CF

#### 3.6.1 Stable phases

At stable phases, regarding germs classified as physiological bacterial flora in pwCF, only *Stomatococcus* showed an association with increases in IL-1β (median, IQR: 4.0, (3.0, 5.3) vs. 8.9, (7.0, 11.2); p=0.006). None of the germs classified as pathogens was associated with changes in soluble inflammatory markers.

#### 3.6.2 Exacerbation

At exacerbation, although only *Moraxella catarrhalis* was detected in four pwCF, this was significantly associated with higher levels of soluble inflammatory markers like IL-1β, IL-6, IL-8, and MMP-9.

### 3.7 Inflammation markers during stable phases and exacerbation in CF patients in relation to medication

#### 3.7.1 Inhalative antibiotics

**Figure 4:**
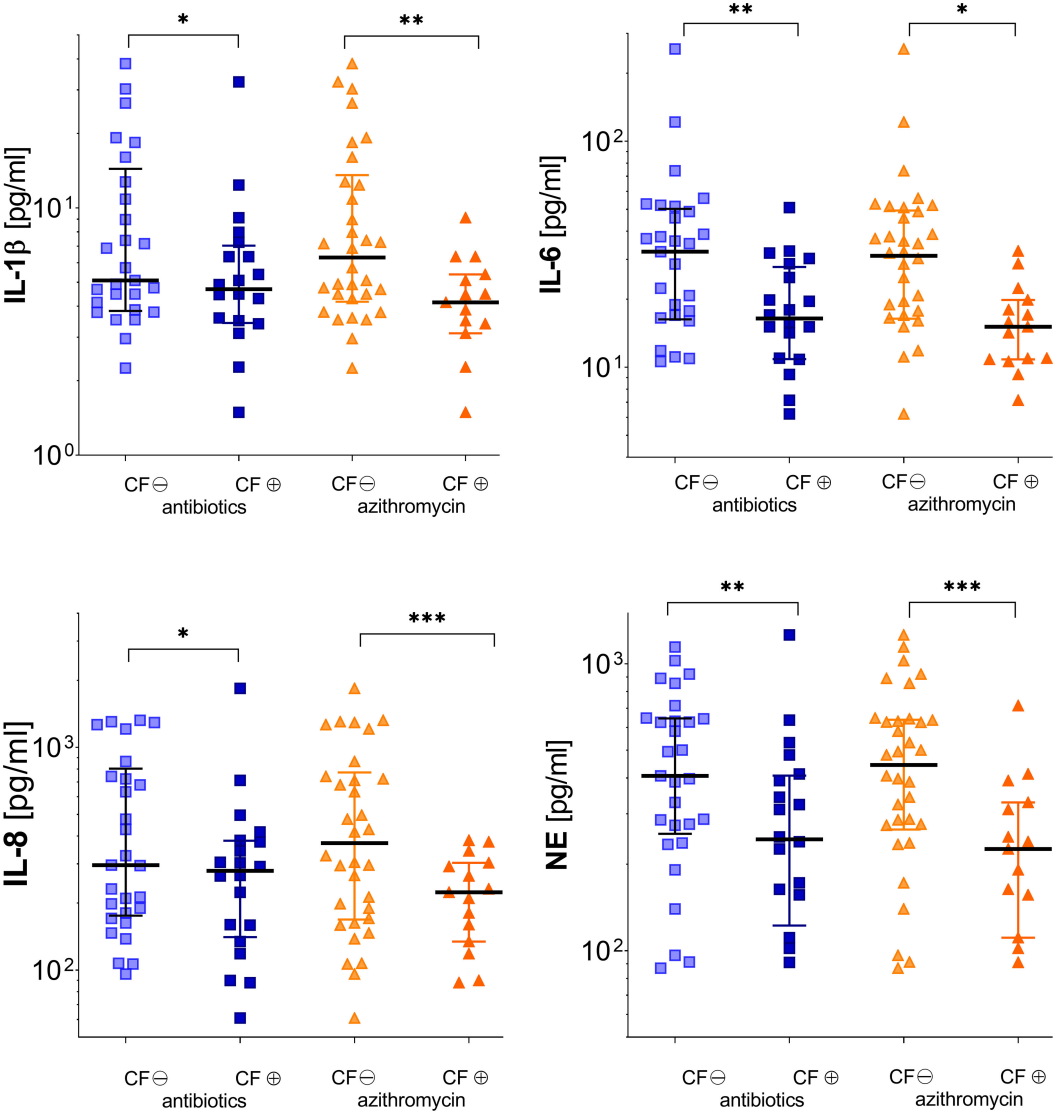
Inflammatory markers in subgroups of patients according to either receiving (⊕) or not receiving (⊖) pulmonary inhaled antibiotics (tobramycin, colistin, and aztreonam) or oral azithromycin (see **Tables 2**, **3**). Significance levels are displayed as follows: *p <0.05; **p<0.01; ***p <0.001.

During stable phases, pwCF regularly inhaling antipseudomonal antibiotics revealed cytokine baseline levels similar to pwCF without such an antibiotic medication. During exacerbation, these patients showed significantly lower levels in pro-inflammatory cytokines like IL-1β, IL-6, and IL-8. Differences between pwCF and healthy controls in IL-8 were, however, mainly driven by the values of five patients who had concentrations above 1,000 µg/ml. NE was also significantly lower than in non-colonized CF patients. Antibiotic therapy did not affect TIMP-1 and MMP-9 level (**Figure 4** and **Table 2**).

**Table 2:** Inflammation markers during stable phase and exacerbation in CF patients in relation to inhalative antibiotics (colistin, tobramycin, or aztreonam).

		Inhalative antibiotics (n=20)		CF patients not inhaling antibiotics (n=29)		p
		median	IQR	median	IQR	
**stable**	IL-1β [pg/ml]	4.3	3.7	4.3	5.0	0.921
	IL-6 [pg/ml]	11.1	15.9	16.3	12.7	0.471
	IL-8 [pg/ml]	198.2	175.2	203.3	283.7	0.828
	NE [pg/ml]	151.1	316.9	269.3	417.0	0.118
	TIMP-1 [pg/ml]	4,738.1	6,024.9	5,254.1	4,603.7	0.305
	MMP-9 [pg/ml]	4,019.3	7,020.1	7,926.3	65,817.0	0.147
**exacerbation**	IL-1β [pg/ml]	4.7	3.6	5.1	10.6	**0.049**
	IL-6 [pg/ml]	16.4	16.9	32.5	33.9	**0.005**
	IL-8 [pg/ml]	278.9	240.3	295.9	627.0	**0.025**
	NE [pg/ml]	244.5	285.4	406.9	389.7	**0.008**
	TIMP-1 [pg/ml]	6,413.3	6,523.8	5,669.0	6,818.0	0.201
	MMP-9 [pg/ml]	7,240.3	15,738.9	10,004.6	75,702.0	0.083

Statistical differences were calculated using the Mann–Whitney U-test. p-values <0.05 were considered statistically significant.

Bold values stand for statistically significant result.

#### 3.7.2 Azythromycin

Therapy with azithromycin only affected significantly MMP-9 levels during stable phase. During exacerbation, patients showed significantly decreased levels in every cytokine measured except TIMP-1 (**Figure 4** and **Table 3**).

**Table 3:** Inflammation markers in CF patients during stable phases and exacerbation in relation to oral medication with azithromycin.

		CF patients receiving azithromycin (n=15)		CF patients not receiving azithromycin (n=34)			
		Median	IQR	median	IQR	p
**stable**	IL-1β [pg/ml]	4.0	2.4	4.4	5.0	0.521
	IL-6 [pg/ml]	11.1	16.0	17.2	12.9	0.218
	IL-8 [pg/ml]	168.2	190.6	204.4	342.7	0.728
	NE [pg/ml]	138.9	209.9	290.6	540.5	0.061
	TIMP-1 [pg/ml]	4,080.2	3,697.7	5,238.9	4,767.6	0.070
	MMP-9 [pg/ml]	3,559.2	6,545.1	7,593.3	54,049.1	**0.042**
**exacerbation**	IL-1β [pg/ml]	4.1	2.3	6.3	9.4	**0.004**
	IL-6 [pg/ml]	15.1	9.0	31.2	33.0	**0.003**
	IL-8 [pg/ml]	223.4	186.6	371.6	602.9	**0.031**
	NE [pg/ml]	226.2	217.8	444.0	373.7	**0.006**
	TIMP-1 [pg/ml]	5,055.0	3,637.8	6,851.0	7,426.4	0.141
	MMP-9 [pg/ml]	7,372.5	6,864.6	10,205.2	69,396.3	**0.016**

Statistical differences were calculated using the Mann–Whitney U-test. p-values <0.05 were considered statistically significant.

Bold values stand for statistically significant result.

Patients simultaneously receiving inhalative antibiotics and azithromycin (n=12) revealed lower baseline levels in all mediators measured except TIMP-1 and MMP-9 and significant lower levels in IL-6 and NE during stable phase and reduced NE levels during exacerbation compared to CF patients only receiving inhalative antibiotics (n=8). Co-medication with both drugs resulted in significantly lower levels in IL-6, IL-8, and NE during stable phase in contrast to medication with azithromycin alone (data not shown). There was no statistically significant change in inflammation markers during stable phase or exacerbation for medication with oral antibiotics or for pulmonary or nasally applied topical steroids.

#### 3.7.3 Comparing inflammation markers of CF patients with and without *S. aureus* colonization during stable phase and during exacerbations

In *S. aureus*-colonized CF patients (n=35), we observed a tendency towards higher levels of IL-6, IL-8, NE, and MMP-9 during stable phases compared to controls. However, the results did not attain statistical significance. During exacerbation, these mediator values tended to be higher in contrast to patients that tested negative for *S. aureus*, without reaching statistical significance. Changes between stable phase and exacerbation in both CF cohorts, i.e. *S. aureus* positive and *S. aureus* negative, were only significant for IL-8 (increase rate, 1.4 and 1.7, respectively; p=0.001) (**Supplementary Table S5**).

#### 3.7.4 Comparing inflammation markers of CF patients with and without *S. aureus* and *P. aeruginosa* co-colonization during stable phases and exacerbations

Patients co-colonized with *S. aureus* and *P. aeruginosa* (n=10) revealed significantly higher baseline levels of IL-6 (increase factor, 2.3; p=0.042), NE (increase factor, 8.4; p=0.003), and MMP-9 (increase factor, 9.2; p=0.022) compared to those only intermittently or permanently colonized with *P. aeruginosa* (n=6). During exacerbation, cytokines reach similar levels in both cohorts; however, NE levels remained elevated in the co-colonized subgroup (increase rate, 3.4; p=0.016). We found no statistical difference between co-colonized patients and CF patients without pathogen colonization or CF patients only colonized with *S. aureus* (**Supplementary Table S6**).

## 4 Discussion

Progressive pulmonary destruction and the resulting respiratory failure after chronic airway colonization and inflammation remain causative for premature death in CF patients. Although UAW and LAW are closely connected, which enables pathogens to descend from the sinuses into the lung and lead to re-infections even after lung transplantation (16, 48), research on inflammatory dynamics in UAW remains underinvestigated. In this study, we investigated soluble inflammatory dynamics in NLF during stable phase and exacerbation in CF patients and healthy controls.

### 4.1 Dynamics in UAW inflammation

CF patients revealed higher baseline levels of inflammatory cytokines during stable phases, and during exacerbation, cytokines did not increase to the level observed in healthy controls. This contributes to previous publications from our group (16, 49) and others (50, 51) showing the chronic inflammatory process typical for CF during stable phase. Nevertheless, cytokine levels during exacerbation were comparable in both groups, and stated differences did not reach statistical significance. In addition, children and adults showed similar inflammatory responses in both groups.

### 4.2 Pathogen detection in UAW

In this study, using multiplex PCR, viral pathogens were significantly more often detected during exacerbatioon/ARI in both cohorts. This is consistent with previous studies reporting viral infections to be responsible for up to 72% of exacerbations in pwCF (25). Furthermore, hRV resulted to be the most frequently detected virus in both CF and HC cohorts. This accords well with previous studies (24, 52–54) in which hRV has been reported as the most prevalent virus also in pwCF. In our study, however, we found that hRV detection rates in pwCF and HCs were similar during stable phases (18.4%), whereas during exacerbation, these rates were significantly higher in HC than in pwCF (65.8% and 22.4%, respectively). Discrepancies between our results and those reporting higher incidences of hRV infections in pwCF are likely due to the younger age of the cohorts considered in those studies. In our study, median age for hRV infection at exacerbation was 13.8 and 23.2 years (p=0.018) for the CF and HC cohorts, respectively.

At stable phases, hRV detection led to significantly higher levels of IL-6 and IL-8 in the whole cohort, (i.e. CF + HC), whereas in the hRV-positive subgroup of pwCF, only IL-6 was elevated. On the other hand, in the whole cohort, virus detection during exacerbation was associated with increased levels of IL-1β, IL-6, IL-8, NE, and MMP-9. Increases in IL-6, IL-8, NE, and MMP-9 were also observed in the subgroup of pwCF revealing viral colonization. This effect is likely due to the predominant effect of hRV colonization in our cohort, as its detection led to higher levels of IL-1β, IL-6, IL-8, NE, and MMP-9, whereas none of the other viruses different from hRV affected the levels of any assessed inflammatory parameter.

Host immune responses to hRV have been previously studied *in vitro* and *in vivo* (26, 55). The single-stranded RNA virus is known to infect the airway epithelium by binding to its target receptor, which varies according to the virus’ subtype (56). After infection, the airway epithelium responds by activating different signaling pathways, leading to the release of interleukin IL-8, regulated on activation, normal T cell expressed and secreted (RANTES, also known as CCL5), and granulocyte-macrophage colony-stimulating factor (GM-CSF), which in turn again recruits neutrophils (55). Other studies in pwCF reported associations between hRV infections and elevated IL-6, IL-8, and neutrophil levels (26, 55).

Regarding bacterial pathogen detection, in our study, only *M. catarrhalis* was found to be significantly associated with the elevation of inflammatory mediators in NLF. In a previous study, *Moraxella* species were found to be strongly associated with higher incidences of respiratory viruses in bronchoalveolar lavage fluid samples from pwCF (33). Nevertheless, in our study, detection rates for this bacterium were comparatively low (n=4), and only one of the hRV-positive patients was simultaneously positive for colonization with *M. catarrhalis*. Therefore, we cannot draw any conclusions regarding the previously reported association.

### 4.3 Impact on UAW inflammation and medication

Medication with inhaled antibiotics (colistin, tobramycin, or aztreonam) and oral therapy with azithromycin significantly reduced all inflammatory cytokines and mediators except for TIMP-1 during exacerbation. This is consistent with recent studies indicating benefits of azithromycin with regard to decreased exacerbation rates (57) and lung function decline, visible also in reduced inflammation mediators (21, 36). It is remarkable that both drugs also show effects in the UAW and even in nasal lavage fluids, although they were orally and pulmonary administered. Azithromycin is known as a non-specific inhibitor of the nuclear factor kappa-light-chain-enhancer of activated B cells (NF-kB) pathway, thus acting as an anti-microbial and anti-inflammatory agent (21, 58). Although overall mechanisms remain elusive, it is known that bacterial infections activate NF-kB pathway *via* toll-like receptors and airway epithelial cells, which, in turn, promotes transcription of pro-inflammatory substances like IL-6, IL-8, and interferon gamma (IFN-γ). Elevated IL-8 levels stimulate NE release in PMN. NE in turn is a major inhibitor of TIMP-1. In our study, TIMP-1 levels remained comparable in both groups, indicating reduced NE activity. As TIMP-1 plays an important role in inhibiting airway remodeling, therapy with azithromycin seems to be essential in preventing respiratory endothelial destruction in the UAW of CF patients. Furthermore, co-medication with inhalative antibiotics and azithromycin shows a synergistic effect, significantly reducing IL-6, IL-8, and NE levels also during stable phase. It therefore seems advisable to apply both drugs simultaneously.

### 4.4 UAW inflammation and colonization status

Consistent with age-related colonization patterns of CF patients in different age groups in German and international registries (19), CF children recruited in this study were significantly more often colonized with *S. aureus*, whereas adults were more likely to have a *P. aeruginosa* colonization.

Colonization with *P. aeruginosa* was associated with lower baseline levels of inflammatory cytokines and further mediators like NE and MMP-9. In concordance with previous publications (21, 22), we attribute this to prescribed anti-inflammatory medication with azithromycin, as all patients with chronic *P. aeruginosa* colonization included in this study received azithromycin orally. As azithromycin directly inhibits the NF-κB pathway (59), which plays an important role in upper airway inflammation, reduced cytokine levels can be directly associated with this medication. We should highlight that this effect was more pronounced during the stable phase than during exacerbation.

Colonization of the UAW with *S. aureus* appears to contribute to the inflammatory process in CF to a higher extent than *P. aeruginosa* colonization (under antimicrobial medication), as patients colonized with *S. aureus* tended to show higher inflammatory mediators with significantly higher levels in IL-6, NE, and MMP-9 during the stable phase. This corresponds to previous findings by Janhsen et al. (22). *S. aureus* possesses different virulence factors like staphylococcal protein A that interact directly with tumor necrosis factor-alpha (TNF-α) receptor leading to a massive release of pro-inflammatory cytokines like IL-1β, IL-6, and IL-8 (60). Further staphylococcal proteins are enterotoxins A and B (SEA and SEB) activating T-cell proliferation and increase cytokine production (61). This pro-inflammatory effect seems to be more visible during the stable phase, as we observed comparable overall cytokine levels in both groups during exacerbation. Beside cytokine release, *S. aureus* apparently also promotes PMN degradation, as we observed elevated MMP-9 and NE levels during the stable phase and both mediators are PMN-secreted. Nowadays, it is assumed that PMNs play a major role in LAW immune system. UAW immune system is considered to be mostly IgA triggered (15, 34, 60, 62). However, elevated levels in MMP-9 and NE indicate a participation of PMNs also in UAW.

Although current guidelines do not advise permanent antibiotic medication in CF patients colonized with *S. aureus* (18), *S. aureus* colonization promotes UAW inflammation and contributes to disease progression.

Similar to single colonization with *S. aureus*, co-colonization with *S. aureus* and *P. aeruginosa* shows significantly higher levels of IL-6, NE, and MMP-9 during stable phases, and NE remains elevated during exacerbation. This indicates a dominant influence of *S. aureus* pathogens in co-colonized CF patients. According to Limoli et al. (63), lung function decline and increased exacerbation rates are associated with co-infections in CF patients. CF patients colonized with *P. aeruginosa* are regularly treated with oral azithromycin, a medication that *S. aureus* is known to be resistant to. Higher inflammatory responses in co-infected patients can thus be directly related to less effective medication. Hence, we should consider a more aggressive treatment including anti-staphylococcal antibiotics in co-infected CF patients with clinical lung deterioration.

### 4.5 Limitations

In six samples, microbiology could not be performed due to an insufficient amount of material. Instead, for these samples, multiplex PCR analysis of viral and pneumonia-causing pathogens were conducted. Some subjects in the healthy control cohort were medical students or staff from the Jena University Hospital. In addition, bacterial colonization for the healthy cohort was not assessed, which may have limited the interpretation of results from comparisons with pwCF, as it is known that health workers may be at higher risk of colonization with MSSA and MRSA. However, rates of MRSA colonization are relatively low in our hospital, compared to other countries, so that we do not expect this to be a relevant confounder.

## 5 Conclusion

Our non-invasive method to sample airway surface liquid from the UAW, also in a home-based setting, is promising and allows even day-to-day assessment of bacterial and viral pathogen colonization and of inflammation, e.g. during novel therapeutic options. Thereby, we confirm that oral azithromycin has the benefit of reducing inflammatory mediators in the UAW segment of CF patients during exacerbation. When combined with inhalative antibiotics, these drugs show synergistic effects, reducing inflammation and assumedly preventing morphological damages and airway remodeling.

## Data availability statement

The raw data supporting the conclusions of this article will be made available by the authors, without undue reservation.

## Ethics statement

The studies involving human participants were reviewed and approved by Prof. Dr. med. Ulrich Brand and Dr. phil. Ulrike Skorsetz, Universitätsklinikum Jena Ethik-Kommission, Bachstraße 18, 07743 Jena, Germany. Written informed consent to participate in this study was provided by the participants’ legal guardian/next of kin.

## Author contributions

JM and JH designed the study. TS performed material preparation and data collection. MB performed conventional microbiological analyses. TA and PB performed multiplex PCRs. NE and TS performed data analyses. CZ and TL gave statistical advice. NE wrote the first draft of the manuscript, supported by JM, CZ, and FD, and all authors commented on previous versions. All authors read and approved the final manuscript.

## Acknowledgments

The authors especially thank all patients and other participants who made this study possible. Special thanks go to Christoph Krüger for his useful IT support and Dr. Christin Arnold and Dr. Anke Jaudszus for contributing to data processing and discussion.

## Conflict of interest

The authors declare that the research was conducted in the absence of any commercial or financial relationships that could be construed as a potential conflict of interest.

## Publisher’s note

All claims expressed in this article are solely those of the authors and do not necessarily represent those of their affiliated organizations, or those of the publisher, the editors and the reviewers. Any product that may be evaluated in this article, or claim that may be made by its manufacturer, is not guaranteed or endorsed by the publisher.

## References

[1] Kerem B Rommens JM Buchanan JA Markiewicz D Cox TK Chakravarti A. Identification of the cystic fibrosis gene: Genetic analysis. Science (1989) 245(4922):1073–80. doi: 10.1126/science.2570460 2570460

[2] Ratjen F Döring G. Cystic fibrosis. Lancet (2003) 361:681–9. doi: 10.1016/S0140-6736(03)12567-6 12606185

[3] Elborn JS. Cystic fibrosis. Lancet (2016) 388:2519–31. doi: 10.1016/S0140-6736(16)00576-6 27140670

[4] Chin M Aaron SD Bell SC. The treatment of the pulmonary and extrapulmonary manifestations of cystic fibrosis. La Presse Médicale (2017) 46:e139–e64. doi: 10.1016/j.lpm.2016.11.030 28576636

[5] O´Sullivan BP Freedman SD. Cystic fibrosis. Lancet (2009) 373:1891–904. doi: 10.1016/S0140-6736(09)60327-5 19403164

[6] Stoltz DA Meyerholz DK Welsh MJ. Origins of cystic fibrosis lung disease. N Engl J Med (2015) 372:351–62. doi: 10.1056/NEJMra1300109 PMC4916857 25607428

[7] Surette MG. The cystic fibrosis lung microbiome. Ann Am Thorac Soc (2014) 11 Suppl 1:S61–5. doi: 10.1513/AnnalsATS.201306-159MG 24437409

[8] Gysin C Alothman GA Papsin BC. Sinonasal disease in cystic fibrosis: Clinical characteristics, diagnosis, and management. Pediatr Pulmonology (2000) 30:481–9. doi: 10.1002/1099-0496(200012)30:6<481::AID-PPUL8>3.0.CO;2-N 11109061

[9] Mauch RM Rossi CL Aiello TB Ribeiro JD Ribeiro AF Høiby N. Secretory iga response against *Pseudomonas aeruginosa* in the upper airways and the link with chronic lung infection in cystic fibrosis. Pathog Dis (2017) 75. doi: 10.1093/femspd/ftx069 28645157

[10] Downey DG Bell SC Elborn JS. Neutrophils in cystic fibrosis. Thorax (2009) 64:81–8. doi: 10.1136/thx.2007.082388 19103874

[11] Conese M Copreni E Gioia SD Rinaldis PD Fumarulo R. Neutrophil recruitment and airway epithelial cell involvement in chronic cystic fibrosis lung disease. J Cystic Fibrosis (2003) 2:129–35. doi: 10.1016/S1569-1993(03)00063-8 15463861

[12] Cantin AM Hartl D Konstan MW Chmiel JF. Inflammation in cystic fibrosis lung disease: Pathogenesis and therapy. J Cystic Fibrosis (2015) 14:419–30. doi: 10.1016/j.jcf.2015.03.003 25814049

[13] Gaggar A Li Y Weathington N Winkler M Kong M Jackson P. Matrix metalloprotease-9 dysregulation in lower airway secretions of cystic fibrosis patients. Am J Physiology-Lung Cell Mol Physiol (2007) 293:L96–L104. doi: 10.1152/ajplung.00492.2006 17384080

[14] Papayannopoulos V Zychlinsky A. Nets: A new strategy for using old weapons. Trends Immunol (2009) 30:513–21. doi: 10.1016/j.it.2009.07.011 19699684

[15] Johansen HK Aanaes K Pressler T Nielsen KG Fisker J Skov M. Colonisation and infection of the paranasal sinuses in cystic fibrosis patients is accompanied by a reduced pmn response. J Cystic Fibrosis (2012) 11:525–31. doi: 10.1016/j.jcf.2012.04.011 22595452

[16] Mainz JG Naehrlich L Schien M Kading M Schiller I Mayr S. Concordant genotype of upper and lower airways *P aeruginosa* and *S aureus* isolates in cystic fibrosis. Thorax (2009) 64(6):535–40. doi: 10.1136/thx.2008.104711 19282318

[17] Ahlgren HG Benedetti A Landry JS Bernier J Matouk E Radzioch D. Clinical outcomes associated with *Staphylococcus aureus* and *Pseudomonas aeruginosa* airway infections in adult cystic fibrosis patients. BMC Pulmonary Med (2015) 15. doi: 10.1186/s12890-015-0062-7 PMC4475617 26093634

[18] Smyth AR Bell SC Bojcin S Bryon M Duff A Flume P. European Cystic fibrosis society standards of care: Best practice guidelines. J Cyst Fibros (2014) 13 Suppl 1:S23–42. doi: 10.1016/j.jcf.2014.03.010 24856775

[19] Foundation CF. Patient registry annual data report (2019). Available at: https://www.cff.org/Research/Researcher-Resources/Patient-Registry/.

[20] Høiby N. Recent advances in the treatment of *Pseudomonas aeruginosa* infections in cystic fibrosis. BMC Med (2011) 9:32. doi: 10.1186/1741-7015-9-32 21463524 PMC3087692

[21] Saiman L Marshall BC Mayer-Hamblett N Burns JL Quittner AL Cibene DA. Azithromycin in patients with cystic fibrosis chronically infected with *Pseudomonas aeruginosa* . JAMA (2003) 290:1749. doi: 10.1001/jama.290.13.1749 14519709

[22] Janhsen WK Arnold C Hentschel J Lehmann T Pfister W Baier M. Colonization of CF patients' upper airways with s. aureus contributes more decisively to upper airway inflammation than p. aeruginosa. Med Microbiol Immunol (2016) 205:485–500. doi: 10.1007/s00430-016-0463-0 27377929

[23] Asner S Waters V Solomon M Yau Y Richardson SE Grasemann H. Role of respiratory viruses in pulmonary exacerbations in children with cystic fibrosis. J Cyst Fibros (2012) 11(5):433–9. doi: 10.1016/j.jcf.2012.04.006 PMC7105203 22579414

[24] Brestovac B Lawrence C Speers DJ Sammels LM Mulrennan S. Respiratory viral infections in Western Australians with cystic fibrosis. Respir Med (2020) 161:105854. doi: 10.1016/j.rmed.2019.105854 32056728

[25] Esposito S Dacco V Daleno C Gambazza S Montinaro V Bisogno A. Human rhinovirus infection in children with cystic fibrosis. Jpn J Infect Dis (2014) 67(5):399–401. doi: 10.7883/yoken.67.399 25241695

[26] Ling KM Garratt LW Lassmann T Stick SM Kicic A Waerp. Elucidating the interaction of CF airway epithelial cells and rhinovirus: Using the host-pathogen relationship to identify future therapeutic strategies. Front Pharmacol (2018) 9:1270. doi: 10.3389/fphar.2018.01270 30464745 PMC6234657

[27] Ling KM Garratt LW Gill EE Lee AHY Agudelo-Romero P Sutanto EN. Rhinovirus infection drives complex host airway molecular responses in children with cystic fibrosis. Front Immunol (2020) 11:1327. doi: 10.3389/fimmu.2020.01327 32765492 PMC7378398

[28] Hendricks MR Bomberger JM. Digging through the obstruction: Insight into the epithelial cell response to respiratory virus infection in patients with cystic fibrosis. J Virol (2016) 90(9):4258–61. doi: 10.1128/JVI.01864-15 PMC4836329 26865718

[29] Kiedrowski MR Bomberger JM. Viral-bacterial Co-infections in the cystic fibrosis respiratory tract. Front Immunol (2018) 9:3067. doi: 10.3389/fimmu.2018.03067 30619379 PMC6306490

[30] Flight W Jones A. The diagnosis and management of respiratory viral infections in cystic fibrosis. Expert Rev Respir Med (2017) 11(3):221–7. doi: 10.1080/17476348.2017.1288102 28132571

[31] de Almeida MB Zerbinati RM Tateno AF Oliveira CM Romao RM Rodrigues JC. Rhinovirus c and respiratory exacerbations in children with cystic fibrosis. Emerg Infect Dis (2010) 16(6):996–9. doi: 10.3201/eid1606.100063 PMC3086221 20507756

[32] Wat D Gelder C Hibbitts S Cafferty F Bowler I Pierrepoint M. The role of respiratory viruses in cystic fibrosis. J Cyst Fibros (2008) 7(4):320–8. doi: 10.1016/j.jcf.2007.12.002 PMC7105190 18255355

[33] Esther CR Jr. Lin FC Kerr A Miller MB Gilligan PH. Respiratory viruses are associated with common respiratory pathogens in cystic fibrosis. Pediatr Pulmonol (2014) 49(9):926–31. doi: 10.1002/ppul.22917 24167159

[34] Fischer N Hentschel J Markert UR Keller PM Pletz MW Mainz JG. Non-invasive assessment of upper and lower airway infection and inflammation in CF patients. Pediatr Pulmonol (2014) 49(11):1065–75. doi: 10.1002/ppul.22982 24464968

[35] Fuchs HJ Borowitz DS Christiansen DH Morris EM Nash ML Ramsey BW. Effect of aerosolized recombinant human dnase on exacerbations of respiratory symptoms and on pulmonary function in patients with cystic fibrosis. N Engl J Med (1994) 331:637–42. doi: 10.1056/NEJM199409083311003 7503821

[36] Hentschel J Müller U Doht F Fischer N Böer K Sonnemann J. Influences of nasal lavage collection-, processing- and storage methods on inflammatory markers — evaluation of a method for non-invasive sampling of epithelial lining fluid in cystic fibrosis and other respiratory diseases. J Immunol Methods (2014) 404:41–51. doi: 10.1016/j.jim.2013.12.003 24370751

[37] Lee TWR Brownlee KG Conway SP Denton M Littlewood JM. Evaluation of a new definition for chronic pseudomonas aeruginosa infection in cystic fibrosis patients. J Cystic Fibrosis (2003) 2:29–34. doi: 10.1016/S1569-1993(02)00141-8 15463843

[38] Aas JA Paster BJ Stokes LN Olsen I Dewhirst FE. Defining the normal bacterial flora of the oral cavity. J Clin Microbiol (2005) 43:5721–32. doi: 10.1128/JCM.43.11.5721-5732.2005 PMC1287824 16272510

[39] Frank DN Feazel LM Bessesen MT Price CS Janoff EN Pace NR. The human nasal microbiota and *Staphylococcus aureus* carriage. PloS One (2010) 5:e10598. doi: 10.1371/journal.pone.0010598 20498722 PMC2871794

[40] Goss CH Burns JL. Exacerbations in cystic fibrosis. 1: Epidemiology and pathogenesis. Thorax (2007) 62(4):360–67. doi: 10.1136/thx.2006.060889 PMC2092469 17387214

[41] Zemanick ET Harris JK Wagner BD Robertson CE Sagel SD Stevens MJ. Inflammation and airway microbiota during cystic fibrosis pulmonary exacerbations. PloS One (2013) 8:e62917. doi: 10.1371/journal.pone.0062917 23646159 PMC3639911

[42] Justicia JL Solé A Quintana-Gallego E Gartner S de Gracia J Prados C. Management of pulmonary exacerbations in cystic fibrosis: Still an unmet medical need in clinical practice. Expert Rev Respir Med (2015) 9:183–94. doi: 10.1586/17476348.2015.1016504 25692532

[43] Hogardt M Häußler S Klingenbiel B Kahl BC Zange S Leitritz L. MiQ 24: Atemwegsinfektionen bei mukoviszidose. In: Qualitätsstandards in der mikrobiologisch-infektiologischen diagnostik. München: Urban & Fischer/Elsevier (2006).

[44] Fehlberg LC Andrade LH Assis DM Pereira RH Gales AC Marques EA. Performance of maldi-tof Ms for species identification of burkholderia cepacia complex clinical isolates. Diagn Microbiol Infect Dis (2013) 77(2):126–8. doi: 10.1016/j.diagmicrobio.2013.06.011 23891221

[45] Puppe W Weigl J Grondahl B Knuf M Rockahr S von Bismarck P. Validation of a multiplex reverse transcriptase pcr Elisa for the detection of 19 respiratory tract pathogens. Infection (2013) 41(1):77–91. doi: 10.1007/s15010-012-0298-6 22847627 PMC7100787

[46] O'Keeffe AG Ambler G Barber JA. Sample size calculations based on a difference in medians for positively skewed outcomes in health care studies. BMC Med Res Methodol (2017) 17(1):157. doi: 10.1186/s12874-017-0426-1 29197347 PMC5712177

[47] Fokkens WJ Lund VJ Mullol J Bachert C Alobid I Baroody F. Epos 2012: European position paper on rhinosinusitis and nasal polyps 2012. A summary for otorhinolaryngologists. Rhinology J (2012) 50:1–12. doi: 10.4193/Rhino12.000 22469599

[48] Mainz JG Hentschel J Schien C Cramer N Pfister W Beck JF. Sinonasal persistence of *Pseudomonas aeruginosa* after lung transplantation. J Cyst Fibros (2012) 11(2):158–61. doi: 10.1016/j.jcf.2011.10.009 22133899

[49] Beiersdorf N Schien M Hentschel J Pfister W Markert UR Mainz JG. Soluble inflammation markers in nasal lavage from CF patients and healthy controls. J Cyst Fibros (2013) 12(3):249–57. doi: 10.1016/j.jcf.2012.08.015 22990051

[50] Armstrong D Grimwood K Carlin JB Carzino R Hull J Olinsky A. Severe viral respiratory infections in infants with cystic fibrosis. Pediatr Pulmonol (1998) 26(6):371–9. doi: 10.1002/(sici)1099-0496(199812)26:6<371::aid-ppul1>3.0.co;2-n 9888211

[51] Armstrong DS Hook SM Jamsen KM Nixon GM Carzino R Carlin JB. Lower airway inflammation in infants with cystic fibrosis detected by newborn screening. Pediatr Pulmonol (2005) 40(6):500–10. doi: 10.1002/ppul.20294 16208679

[52] Loughlin R Wilbur JD McNally FJ Nedelec FJ Heald R. Katanin contributes to interspecies spindle length scaling in xenopus. Cell (2011) 147(6):1397–407. doi: 10.1016/j.cell.2011.11.014 PMC3240848 22153081

[53] Burns JL Emerson J Kuypers J Campbell AP Gibson RL McNamara S. Respiratory viruses in children with cystic fibrosis: Viral detection and clinical findings. Influenza Other Respir Viruses (2012) 6(3):218–23. doi: 10.1111/j.1750-2659.2011.00292.x PMC4941093 21955319

[54] Dijkema JS van Ewijk BE Wilbrink B Wolfs TF Kimpen JL van der Ent CK. Frequency and duration of rhinovirus infections in children with cystic fibrosis and healthy controls: A longitudinal cohort study. Pediatr Infect Dis J (2016) 35(4):379–83. doi: 10.1097/INF.0000000000001014 26658528

[55] Jacobs SE Lamson DM St George K Walsh TJ. Human rhinoviruses. Clin Microbiol Rev (2013) 26(1):135–62. doi: 10.1128/CMR.00077-12 PMC3553670 23297263

[56] Vandini S Biagi C Fischer M Lanari M. Impact of rhinovirus infections in children. Viruses (2019) 11(6). doi: 10.3390/v11060521 PMC6632063 31195744

[57] Saiman L Anstead M Mayer-Hamblett N Lands LC Kloster M Hocevar-Trnka J. Effect of azithromycin on pulmonary function in patients with cystic fibrosis uninfected with *Pseudomonas aeruginosa*: A randomized controlled trial. JAMA (2010) 303(17):1707–15. doi: 10.1001/jama.2010.563 20442386

[58] Bell SC Senini SL McCormack JG. Macrolides in cystic fibrosis. Chron Respir Dis (2005) 2(2):85–98. doi: 10.1191/1479972305cd066rs 16279156

[59] Stellari FF Caruso P Topini T Carnini C Facchinetti F Villetti G. Anti-inflammatory effects of azithromycin evaluated by *in vivo* imaging of nf-kb activation in a mouse model of acute lung inflammation. In: D30 novel approaches to assessing lung pathophysiology. New York, USA: American Thoracic Society (2012). p. A5585–A.

[60] Gomez MI Lee A Reddy B Muir A Soong G Pitt A. Staphylococcus aureus protein a induces airway epithelial inflammatory responses by activating Tnfr1. Nat Med (2004) 10(8):842–8. doi: 10.1038/nm1079 15247912

[61] Huvenne W Hellings PW Bachert C. Role of staphylococcal superantigens in airway disease. Int Arch Allergy Immunol (2013) 161(4):304–14. doi: 10.1159/000350329 23689556

[62] Doht F Hentschel J Fischer N Lehmann T Markert UR Boer K. Reduced effect of intravenous antibiotic treatment on sinonasal markers in pulmonary inflammation. Rhinology (2015) 53(3):249–59. doi: 10.4193/Rhin14.300 26363166

[63] Limoli DH Yang J Khansaheb MK Helfman B Peng L Stecenko AA. *Staphylococcus aureus* and *Pseudomonas aeruginosa* Co-infection is associated with cystic fibrosis-related diabetes and poor clinical outcomes. Eur J Clin Microbiol Infect Dis (2016) 35(6):947–53. doi: 10.1007/s10096-016-2621-0 26993289

