# Supplementary material for: Divergent dynamics of inflammatory mediators and multiplex PCRs during airway infection in cystic fibrosis patients and healthy controls: Serial upper airway sampling by nasal lavage
Source: Front Immunol. 2022 Nov 18;13:947359. doi: 10.3389/fimmu.2022.947359 (PMC9716083; doi:10.3389/fimmu.2022.947359)
Supplement: Supplementary file 1 [file Table_1.docx]

***Supplementary Material***

1. **Tables**

**Table S1.** Colonization status of UAW and LAW and prescribed antibiotic therapy of the included CF patients. P. aeruginosa: Pseudomonas aeruginosa, S. aureus: Staphylococcus aureus

|  | **Total CF patients** | | | | | **Paediatric CF cohort** | | **Adult CF cohort** | |
| --- | --- | --- | --- | --- | --- | --- | --- | --- | --- |
| **Colonization status of *P. aeruginosa*** | | |  | |  | |  |  |  |
| *UAW* intermittent | | 9/49 | 18.4% | | 6/30 | | 20.0% | 3/19 | 15.7% |
| *UAW* chronic | | 7/49 | 14.0% | | 0/30 | | 0.0% | 7/19 | 36.8% |
| *LAW intermittent* | | 9/49 | 18.4% | | 7/30 | | 23.3% | 2/19 | 10.5% |
| *LAW chronic* | | 12/49 | 24.4% | | 2/30 | | 6.0% | 10/19 | 52.6% |
| *UAW and LAW chronic* | | 7/49 | 14.2% | | 7/30 | | 23.3% | 0/19 | 0.0% |
| **Colonization status of *S. aureus* (MSSA/MRSA)** | | | | |  | |  |  |  |
| UAW intermittent | | 11/49 | 22.4% | | 7/30 | | 23.3% | 4/19 | 21.0% |
| UAW chronic | | 24/49 | 49.0% | | 18/30 | | 60.0% | 6/19 | 31.5% |
| LAW intermittent | | 11/49 | 22.4% | | 7/30 | | 23.3% | 4/19 | 21.0% |
| LAW chronic | | 27/49 | 55.1% | | 19/30 | | 63.3% | 8/19 | 42.1% |
| UAW and LAW chronic | | 22/49 | 44.9% | | 16/30 | | 53.3% | 6/19 | 31.5% |
| **Co-colonization (*S. aureus* + *P. aeruginosa*)** | | | | |  | |  |  |  |
| UAW | | 10/49 | | 20.4% | 6/30 | | 20.0% | 4/19 | 21.1% |
| LAW | | 13/49 | 26.5% | | 8/30 | | 25.7% | 5/19 | 26.3% |
| **Antibiotic therapy** | |  |  | |  | |  |  |  |
| Oral intermittent  (<1- 10 months) | | 18/49 | 36.7% | | 15/30 | | 50.0% | 3/19 | 15.7% |
| Oral permanent (non-azithromycin) | | 5/49 | 10.2% | | 1/30 | | 3.3% | 4/19 | 21.1% |
| pulmonal inhalation | | 20/49 | 40.8% | | 10/30 | | 33.3% | 10/19 | 52.6% |
| Azithromycin oral | | 16/49 | 32.7% | | 5/30 | | 16.6% | 11/19 | 57.9% |
|  | |  |  | |  | |  |  |  |

**Table S2.** Metric and ordinal variables of included CF patients and healthy controls. BMI: Body mass index, BMI-Perc.: BMI-Percentile, FEV1: forced expiratory volume in 1 s, MEF 75/25: mean expiratory flow at 75/25% vital capacity, SD: standard deviation

|  | **Total CF patients** | | | | **Paediatric CF cohort** | | | **Adult CF cohort** | | |
| --- | --- | --- | --- | --- | --- | --- | --- | --- | --- | --- |
|  | Mean ± SD | Median | Range | | Mean ± SD | Median | Range | Mean ± SD | Median | Range |
| Weight (kg) | 43.4 ± 19.0 | 49.0 | 11.6 - 84.1 | | 33.5 ± 16.5 | 28.0 | 11.6 - 64.2 | 59.1 ± 10.3 | 60.1 | 44.3 - 84.1 |
| Height (cm) | 148.9 ± 26.4 | 160 | 89.0 – 186.0 | | 136.1±25.9 | 25.9 | 89-184 | 169.0 ± 8.8 | 170.0 | 156 - 186 |
| BMI (kg/m^2^)  (BMI-Perc.) |  |  |  | | 16.8 ± 3.0  (36.0 ± 23.5) | 15.63  33.7 | 13.3 - 25.4  (2.3 - 91.8) | 20.6 ± 2.7 | 20.8 | 15.1 - 25.4 |
| ESR after 30 min (mm/h) | 14.8 ± 13.9 | 8.0 | 3.0 – 55.0 | | 7.7 ± 5.3 | 5.0 | 3.0 – 19.0 | 22.5 ± 16.2 | 18.0 | 4.0 – 55.0 |
| FEV1 (l)/ (% predicted)  stable | n=42  2.2 ± 1.1  (79.6 ±30.9) | 1.97 (88.8) | 0.5 - 4.9  (21.5 - 125.7) | | n=23  2.1 ± 0.7  (93.4 ± 22.1) | 2.0  (94.4) | 0.5 - 3.6  (42.1 - 125.7) | n=19  2.3 ± 1.4  (63.0 ±31.9) | 2.0  57.0 | 0.7 - 4.9  (21.5 -114.0) |
| MEF75/25(l)/ %predicted)  stable | n=42  2.0 ± 1.2  (61.3 ±35.9) | 2.0  (68.8) | 0.3 - 5.6  (7.5 - 114.6) | | n=23  2.2 ± 0.8  (80.3 ± 26.2) | 2.3  (88.2) | 0.7 - 3.6  (17.5 - 113.9) | n=19  1.7 ±1.5  (38.4 ±32.5) | 1.1  (25.9) | 0.3 - 5.6  (7.5 - 114.6) |
| FEV1 (l)/ (%predicted)  exacerbation | n=35  2.06 ± 1.02  (72.3 ±30.2) | 1.8  (83.8) | 0.7-4.7  (22.9 - 116.8) | | n=18  2.1 ± 0.7  78.6 ± 23.1 | 1.9  (89.1) | 0.8 - 3.5  (27.4 - 116.8) | n=17  1.5 ± 1.3  (45.9 ±30.1) | 1.7  (45.9) | 0.7 - 4.7  (22.9 -110.8) |
| MEF75/25(l)/ %predicted)  exacerbation | n=81  1.7 ± 1.2  (52.3 ±34.4) | 1.4  (57.3) | 0.3 - 5.6  (8.2 - 126.2) | | n=43  1.9 ± 0.9  (67.8 ± 27.7) | 1.9  (67.8) | 0.4 - 3.6  (8.2 - 126.2) | n=38  1.5±1.5  (33.9±32.5) | 0.8  (17.6) | 0.3 - 5.6  (9.1 - 114.7) |
| **Healthy controls** | | | | **Paediatric controls** | | | | **Adult controls** | | |
| Weight (kg) | 51.1 ± 25.0 | 58.0 | 10.0 – 101.0 | | 19.7 - 10.6 | 17.0 | 10.0 - 48.2 | 66.1 ± 13.2 | 63.0 | 46.0 - 101.0 |
| Height (cm) | 152.4 ± 32.5 | 166.0 | 80.0 – 173.0 | | 111.3 ± 25.0 | 108.0 | 80.0 – 167.0 | 172.1 ± 8.0 | 173.0 | 158.0 - 193.0 |
| BMI (kg/m^2^)  (BMI-perc.%) |  |  |  | | 15.0 ± 1.7  (21.2 ± 26.5) | 14.5  (9.7) | 13.6 ± 18.8  (2.9 ± 94.5) | 22.2 ± 3.2 | 21.2 | 16.9 ± 31.9 |

**Table S3.** Inflammation markers in CF and control cohort during stable phase and exacerbation p values were calculated using Mixed ANOVA. If normality assumption was not met (as in IL-1β and IL8 cases), tests were performed on log10- transformed data parameters. Phase effect p values refer to statistical significance for within-subject changes, i.e. changes occurring between stable phases and exacerbations in both cohorts. Cohort effect p values refers to statistical significance for between-subject differences, i.e. differences between means of CF and healthy cohorts. P values labelled as phase*cohort represent statistical significance for differences in the within-subject trends between both subgroups. P values corresponding to pairwise comparisons are not included.

|  | **CF cohort** | | **Healthy cohort** | | |  |  |  |
| --- | --- | --- | --- | --- | --- | --- | --- | --- |
|  | **stable** | **exacerbation** | **stable** | | **ARI** | **phase effect (p)** | **cohort effect (p)** | **phase*cohort (p)** |
| **IL-1β (pg/ml)**  Median  IQR | 4.3  3.9 | 4.9  5.4 | 4.8  5.4 | 9.2  8.8 | | **<0.001** | 0.965 | 0.095 |
| **IL-6 (pg/ml)**  Median  IQR | 15.7  11.5 | 19.8  24.4 | 8.7  8.4 | 28.4  61.7 | | **0.001** | 0.126 | **0.047** |
| **IL-8 (pg/ml)**  Median  IQR | 203.3  194.4 | 294.6  493.8 | 143.7  221.0 | 327.9  699.5 | | **<0.001** | 0.762 | 0.069 |
| **NE (pg/ml)**  Median  IQR | 224.9  388.7 | 328.9  443.8 | 233.8  345.7 | 372.8  359.0 | | **0.027** | 0.977 | 0.205 |
| **TIMP1 (pg/ml)**  Median  IQR | 4890.9  4705.5 | 6404.3  6268.1 | 6165.5  6327.0 | 6083.0  4842.6 | | 0.131 | 0.678 | 0.394 |
| **MMP9 (pg/ml)**  Median  IQR | 5444.8  11752.5 | 7589.0  34000.8 | 5533.8  16916.9 | 24248.2  49890.2 | | **<0.001** | 0.973 | 0.188 |

**Table S4.** Inflammation markers in CF and control cohort for children and adults during stable phase and exacerbation. p values were calculated using Mixed ANOVA. If normality assumption was not met (as in IL-1β and IL8 cases), tests were performed on log10- transformed data parameters. Phase effect p values refer to statistical significance for within-subject changes, i.e. changes occurring between stable phases and exacerbations in both cohorts. Cohort effect p values refers to statistical significance for between subject differences, i.e. differences between means of CF and healthy cohorts. P values labelled as phase*cohort*age represent statistical significance for differences in the within-subject trends between CF, healthy control and paediatric subgroups.

|  | **CF child**  n=30 | | **CF adult**  n=19 | | **Paediatric healthy cohort**  n= 14 | | **Adult healthy cohort**  n=24 | | **phase**  **effect**  (p) | **cohort**  **effect**  (p) | **phase***  **cohort*age**  (p) |
| --- | --- | --- | --- | --- | --- | --- | --- | --- | --- | --- | --- |
|  | stable | exacer-bation | stable | exacer-  bation | stable | ARI | stable | ARI |  |  |  |
| **IL-1ß [pg/ml]** |  |  |  |  |  |  |  |  |  |  |  |
| Median | 4.5 | 5.6 | 4.0 | 3.9 | 6.3 | 12.7 | 4.6 | 7.9 | **<0.001** | 0.144 | 0.292 |
| IQR | 6.3 | 6.8 | 3.4 | 3.0 | 12.3 | 43.6 | 6.7 | 8.6 |  |  |  |
| **IL-6 [pg/ml]** |  |  |  |  |  |  |  |  |  |  |  |
| Median | 17.2 | 21.6 | 11.9 | 16.1 | 9.4 | 25.5 | 8.7 | 30.2 | **0.005** | 0.193 | 0.13 |
| IQR | 13.0 | 20.2 | 9.3 | 38.1 | 20.4 | 32.9 | 6.4 | 71.4 |  |  |  |
| **IL-8 [pg/ml]** |  |  |  |  |  |  |  |  |  |  |  |
| Median | 215.3 | 315.6 | 168.2 | 209.8 | 240.7 | 356.6 | 121.3 | 308.1 | **<0.001** | 0.95 | 0.298 |
| IQR | 300.3 | 490.5 | 225.0 | 257.6 | 240.1 | 1774.8 | 180.3 | 622.5 |  |  |  |
| **NE [pg/ml]** |  |  |  |  |  |  |  |  |  |  |  |
| Median | 290.6 | 447.2 | 118.8 | 237.4 | 268.3 | 304.3 | 166.3 | 420.0 | 0.063 | 0.126 | 0.238 |
| IQR | 515.2 | 370.7 | 231.8 | 286.5 | 336.7 | 340.1 | 364.4 | 356.0 |  |  |  |
| **TIMP-1 [pg/ml]** |  |  |  |  |  |  |  |  |  |  |  |
| Median | 5081.1 | 6453.9 | 4267.0 | 5150.0 | 4782.6 | 4794.4 | 6808.3 | 7180.9 | 0.260 | 0.127 | 0.179 |
| IQR | 4373.1 | 8105.5 | 5561.9 | 6056.7 | 3521.4 | 2517.6 | 5787.9 | 5742.1 |  |  |  |
| **MMP9 [pg/ml]** |  |  |  |  |  |  |  |  |  |  |  |
| Median | 6168.8 | 10205.2 | 4479.4 | 7372.5 | 16950.0 | 35938.9 | 3389.3 | 12967.1 | **<0.001** | 0.799 | 0.768 |
| IQR | 55019.2 | 69455.0 | 7424.1 | 8973.9 | 38529.8 | 71883.4 | 5374.5 | 41483.8 |  |  |  |

**Table S5.** Soluble inflammatory parameters in *S.aureus* positive and negative CF patients during stable phase and exacerbation. p-values were calculated using Mixed ANOVA. If normality assumption was not met (as in IL-1β and IL8 cases), tests were performed on log10- transformed data parameters. Phase effect p values refer to statistical significance for within-subject changes, i.e. changes occurring between stable phases and exacerbations in both cohorts. Cohort effect p values refers to statistical significance for between subject differences, i.e. differences between means of CF and healthy cohorts. P values labelled as phase*cohort represent statistical significance for differences in the within-subject trends between both subgroups.

|  | ***S.aureus* positive CF**  n=35 | | ***S. aureus* negative CF**  n=14 | | **phase**  **effect**  (p) | **cohort effect**  (p) | **phase * cohort**  (p) |
| --- | --- | --- | --- | --- | --- | --- | --- |
|  | **stable** | **exacerbation** | **stable** | **exacerbation** |  |  |  |
| **IL-1ß [pg/ml]** |  |  |  |  |  |  |  |
| Median | 4.5 | 5.4 | 3.9 | 4.7 | 0.087 | 0.140 | 0.903 |
| IQR | 4.3 | 8.6 | 2.8 | 3.7 |  |  |  |
| **IL6 [pg/ml]** |  |  |  |  |  |  |  |
| Median | 16.3 | 20.8 | 9.7 | 17.0 | **0.004** | 0.581 | **0.037** |
| IQR | 12.1 | 23.6 | 16.3 | 26.0 |  |  |  |
| **IL8 [pg/ml]** |  |  |  |  |  |  |  |
| Median | 204.4 | 295.9 | 127.0 | 210.9 | **0.001** | 0.420 | 0.255 |
| IQR | 244.1 | 507.9 | 240.0 | 559.4 |  |  |  |
| **NE [pg/ml]** |  |  |  |  |  |  |  |
| Median | 260.3 | 343.0 | 113.0 | 319.8 | 0.220 | 0.273 | 0.227 |
| IQR | 433.7 | 375.9 | 309.0 | 484.1 |  |  |  |
| **TIMP1 [pg/ml]** |  |  |  |  |  |  |  |
| Median | 5223.7 | 6404.3 | 4384.7 | 5785.9 | 0.124 | 0.284 | 0.804 |
| IQR | 4906.8 | 7025.0 | 5198.9 | 6592.7 |  |  |  |
| **MMP9 [pg/ml]** |  |  |  |  |  |  |  |
| Median | 7212.6 | 7834.3 | 1268.1 | 7423.9 | **0.017** | 0.999 | 0.214 |
| IQR | 11796.3 | 24092.2 | 8477.9 | 61751.0 |  |  |  |

**Table S6.** Soluble inflammatory parameters in with S. aureus and P. aeruginosa co-colonized CF patients compared to P. aeruginosa positive CF patients during stable phase and exacerbation. p-values were calculated using Man-Whitney-U-Test

|  | **Co-colonized CF**  ***S.aureus* + *P. aeruginosa***  n=10 | | **CF patients with *P.aeruginosa* colonization without *S.aureus***  n=6 | | p  stable | p  exacer-bation |
| --- | --- | --- | --- | --- | --- | --- |
|  | **stable** | **exacerbation** | **stable** | **exacerbation** |  |  |
| **IL-1ß [pg/ml]** |  |  |  |  |  |  |
| Median | 4.5 | 4.6 | 4.3 | 4.0 | 0.220 | 0.220 |
| IQR | 5.2 | 7.5 | 3.7 | 2.7 |  |  |
| **IL6 [pg/ml]** |  |  |  |  |  |  |
| Median | 19.5 | 19.7 | 8.5 | 13.9 | **0.042** | 0.220 |
| IQR | 19.3 | 17.7 | 11.5 | 21.0 |  |  |
| **IL8 [pg/ml]** |  |  |  |  |  |  |
| Median | 239.5 | 278.9 | 121.0 | 191.7 | 0.220 | 0.313 |
| IQR | 158.0 | 248.1 | 228.6 | 210.0 |  |  |
| **NE [pg/ml]** |  |  |  |  |  |  |
| Median | 521.9 | 464.3 | 62.1 | 137.4 | **0.003** | **0.016** |
| IQR | 705.2 | 400.3 | 99.7 | 173.0 |  |  |
| **TIMP1 [pg/ml]** |  |  |  |  |  |  |
| Median | 6072.1 | 5807.5 | 3135.9 | 5102.3 | 0.118 | 0.875 |
| IQR | 4570.5 | 8469.4 | 3368.2 | 3304.0 |  |  |
| **MMP9 [pg/ml]** |  |  |  |  |  |  |
| Median | 10595.9 | 14019.0 | 1151.2 | 7423.9 | **0.022** | 0.263 |
| IQR | 52909.5 | 47445.1 | 3947.6 | 6610.8 |  |  |

**Table S7.** Distribution of nasal lavage (NL) samples during stable phase and each exacerbation time point per cohort.

|  | **Stable phase** | **Exacerbation timepoint 1** | **Exacerbation timepoint 2** | **Exacerbation timepoint 3** | **Exacerbation timepoint 4** | **Total** |
| --- | --- | --- | --- | --- | --- | --- |
| **CF patients** | 49 | 49 | 23 | 3 | 2 | 126 |
| **Healthy controls** | 38 | 38 | 9 | 2 | 1 | 88 |
| **Total** | 87 | 87 | 32 | 5 | 3 | 214 |

**Table S8.** Bacterial, fungal and viral pathogens detected by PCR per cohort during stable phase and each exacerbation time point (t_1_, t_2_, t_3_ and t_4_).

|  |  | **Stable phase** | | | **Exacerbation t_1_** | | | **Exacerbation t_2_** | | | **Exacerbation t_3_** | | | **Exacerbation t_4_** | | |
| --- | --- | --- | --- | --- | --- | --- | --- | --- | --- | --- | --- | --- | --- | --- | --- | --- |
|  |  | **CF**  **n (%)** | **HC**  **n (%)** | **p** | **CF**  **n (%)** | **HC**  **n (%)** | **p** | **CF**  **n (%)** | **HC**  **n (%)** | **p** | **CF**  **n (%)** | **HC**  **n (%)** | **p** | **CF**  **n (%)** | **HC**  **n (%)** | **p** |
| **Viral pathogens** | hRV | 9 (18.4) | 7 (18.4) | ns | 11 (22.4) | 25 (65.8) | **^††^** | 8 (34.8) | 4 (44.4) | ns | 2 (66.7) | 1 (50) | ^†^ | 0 (0) | 0 (0) | ^†^ |
|  | Adenoviruses | 1 (2) | 3 (7.9) | ^†^ | 2 (4.1) | 1 (2.6) | ^†^ | 1 (4.3) | 1 (11.1) | ^†^ | 0 (0) | 0 (0) | ^†^ | 0 (0) | 0 (0) | ^†^ |
|  | Enteroviruses | 2 (4.1) | 1 (2.6) | ^†^ | 4 (8.2) | 0 (0) | ^†^ | 0 (0) | 0 (0) | ^†^ | 0 (0) | 0 (0) | ^†^ | 0 (0) | 0 (0) | ^†^ |
|  | Parainfluenza^a^ | 0 (0) | 2 (5.3) | ^†^ | 1 (2.0) | 0 (0) | ^†^ | 4 (17.4) | 0 (0) | ^†^ | 0 (0) | 0 (0) | ^†^ | 0 (0) | 0 (0) | ^†^ |
|  | Coronaviruses | 1 (2.0) | 1 (2.6) | ^†^ | 0 (0) | 0 (0) | ^†^ | 0 (0) | 1 (11.1) | ^†^ | 0 (0) | 0 (0) | ^†^ | 0 (0) | 0 (0) | ^†^ |
|  | hMPV | 0 (0) | 0 (0) | ^†^ | 1 (2.0) | 1 (2.6) | ^†^ | 0 (0) | 0 (0) | ^†^ | 0 (0) | 0 (0) | ^†^ | 0 (0) | 0 (0) | ^†^ |
|  |  |  |  |  |  |  |  |  |  |  |  |  |  |  |  |  |
| **Bacterial and fungal pathogens** | Coagulase-negative staphylococci | 32 (65.3) |  |  | 27 (55.1) |  |  | 17 (73.9) |  |  | 1 (33.3) |  |  | 0 (0) |  |  |
|  | α-hemolytic streptococcus | 21 (42.85) |  |  | 13 (26.5) |  |  | 6 (26.1) |  |  | 1 (33.3) |  |  | 0 (0) |  |  |
|  | *Staphylococcus aureus* | 20 (40.8) |  |  | 20 (40.8) |  |  | 8 (34.8) |  |  | 1 (33.3) |  |  | 0 (0) |  |  |
|  | *Corynebacterium* species | 7 (14.28) |  |  | 0 (0) |  |  | 1 (4.3) |  |  | 0 (0) |  |  | 0 (0) |  |  |
|  | *Pseudomonas* species | 6 (12.2) |  |  | 8 (16.3) |  |  | 5 (21.7) |  |  | 1 (33.3) |  |  | 0 (0) |  |  |
|  | *Pseudomonas aeruginosa* | 6 (12.2) |  |  | 9 (18.4) |  |  | 5 (21.7) |  |  | 1 (33.3) |  |  | 0 (0) |  |  |
|  | *Stomatococcus* | 6 (12.2) |  |  | 6 (12.2) |  |  | 0 (0) |  |  | 0 (0) |  |  | 0 (0) |  |  |
|  | *Enterococcus* species | 5 (10.2) |  |  | 1 (2.0) |  |  | 1 (4.3) |  |  | 0 (0) |  |  | 0 (0) |  |  |
|  | *Pseudomonas aeruginosa* mucoid | 3 (6.1) |  |  | 5 (10.2) |  |  | 4 (17.4) |  |  | 0 (0) |  |  | 0 (0) |  |  |
|  | *Neisseria* | 3 (6.1) |  |  | 5 (10.2) |  |  | 2 (8.7) |  |  | 0 (0) |  |  | 0 (0) |  |  |
|  | *non-hemolytic Streptococcus* | 2 (4.1) |  |  | 0 (0) |  |  | 2 (8.7) |  |  | 1 (33.3) |  |  | 0 (0) |  |  |
|  | *Streptococcus pneumoniae* | 2 (4.1) |  |  | 4 (8.2) |  |  | 1 (4.3) |  |  | 0 (0) |  |  | 0 (0) |  |  |
|  | *Haemophilus influenzae* | 2 (4.1) |  |  | 4 (8.2) |  |  | 2 (8.7) |  |  | 0 (0) |  |  | 0 (0) |  |  |
|  | *Proteus* species | 2 (4.1) |  |  | 2 (4.1) |  |  | 0 (0) |  |  | 0 (0) |  |  | 0 (0) |  |  |
|  | *Proteus mirabilis* | 2 (4.1) |  |  | 2 (4.1) |  |  | 0 (0) |  |  | 0 (0) |  |  | 0 (0) |  |  |
|  | *Haemophilus parainfluenzae* | 1 (2.0) |  |  | 3 (6.1) |  |  | 0 (0) |  |  | 0 (0) |  |  | 0 (0) |  |  |
|  | *Escherichia coli* | 1 (2.0) |  |  | 1 (2.0) |  |  | 0 (0) |  |  | 0 (0) |  |  | 0 (0) |  |  |
|  | *Moraxella_*species | 1 (2.0) |  |  | 6 (12.2) |  |  | 0 (0) |  |  | 0 (0) |  |  | 0 (0) |  |  |
|  | *Moraxela catarrhalis* | 1 (2.0) |  |  | 4 (8.2) |  |  | 0 (0) |  |  | 0 (0) |  |  | 0 (0) |  |  |
|  | *Bacillus* species | 1 (2.0) |  |  | 0 (0) |  |  | 0 (0) |  |  | 0 (0) |  |  | 0 (0) |  |  |
|  | *Citrobacter koseri* | 1 (2.0) |  |  | 0 (0) |  |  | 0 (0) |  |  | 0 (0) |  |  | 0 (0) |  |  |
|  | *Ralstonia picketii* | 1 (2.0) |  |  | 0 (0) |  |  | 0 (0) |  |  | 0 (0) |  |  | 0 (0) |  |  |
|  | *Candida* species. | 1 (2.0) |  |  | 2 (4.1) |  |  | 0 (0) |  |  | 1 (33.3) |  |  | 0 (0) |  |  |
|  | *Candida albicans* | 1 (2.0) |  |  | 2 (4.1) |  |  | 0 (0) |  |  | 1 (33.3) |  |  | 0 (0) |  |  |
|  | *Streptococcus pyogenes* | 0 (0) |  |  | 1 (2.0) |  |  | 0 (0) |  |  | 0 (0) |  |  | 0 (0) |  |  |
|  | *Enterobacter aerogenes* | 0 (0) |  |  | 1 (2.0) |  |  | 0 (0) |  |  | 0 (0) |  |  | 0 (0) |  |  |
|  | Enterobact.cloacae | 0 (0) |  |  | 1 (2.0) |  |  | 1 (4.3) |  |  | 0 (0) |  |  | 0 (0) |  |  |
|  | *Achromobacter xylosoxidans* | 0 (0) |  |  | 1 (2.0) |  |  | 0 (0) |  |  | 0 (0) |  |  | 0 (0) |  |  |
|  | *Achromobacter denitrificans* | 0 (0) |  |  | 0 (0) |  |  | 1 (4.3) |  |  | 0 (0) |  |  | 0 (0) |  |  |
|  | *Acinetobacter lwoffii* | 0 (0) |  |  | 0 (0) |  |  | 1 (4.3) |  |  | 0 (0) |  |  | 0 (0) |  |  |
|  | *Acinetobacter baumannii* | 0 (0) |  |  | 2 (4.1) |  |  | 0 (0) |  |  | 0 (0) |  |  | 0 (0) |  |  |
|  | *Cronobacter sakazakii* | 0 (0) |  |  | 1 (2.0) |  |  | 0 (0) |  |  | 0 (0) |  |  | 0 (0) |  |  |
|  | *Pantoea agglomerans* | 0 (0) |  |  | 1 (2.0) |  |  | 0 (0) |  |  | 0 (0) |  |  | 0 (0) |  |  |
|  | *Neurospora crassa* | 0 (0) |  |  | 0 (0) |  |  | 1 (4.347) |  |  | 0 (0) |  |  | 0 (0) |  |  |
|  | *Stenotrophomonas maltophilia* | 0 (0) |  |  | 1 (2.0) |  |  | 0 (0) |  |  | 0 (0) |  |  | 0 (0) |  |  |
|  |  |  |  |  |  |  |  |  |  |  |  |  |  |  |  |  |

hRV: human rhinovirus, hMPV: human metapneumovirus

^a^ Parainfluenza viruses types 1-4

^†^ insufficient number of samples to compute statistical significance

**^††^** p < 0.0001

ns: not statistically significant

|  |  | **pwCF** | |  |  | **HC** | |  |  | **hRV pos.** |  | **hRV neg** |
| --- | --- | --- | --- | --- | --- | --- | --- | --- | --- | --- | --- | --- |
|  |  | **hRV pos.**  (median, IQR) | **hRV neg.**  (median, IQR) | **p** |  | **hRV pos.**  (median, IQR) | **hRV neg.**  (median, IQR) | **p** |  | **CF vs HC**  **p** |  | **CF vs HC**  **p** |
| **IL-1β** [pg/ml] | Stable phase | 4.3  (4.0, 11.2)  n=9 | 4.3  (3.1, 6.4)  n=40 | n.s. |  | 6.3  (2.8, 8.3)  n=7 | 4.6  (0.1, 7.7)  n=31 | n.s. |  | n.s. |  | n.s. |
|  | Exacerbation | 7.3  (4.5, 20.2)  n=11 | 4.4  (3.3, 7.2)  n=38 | * |  | 9.6  (7.5, 16.4)  n=25 | 4.0  (0.02, 9.1)  n=13 | * |  | n.s. |  | n.s. |
| **IL-6** [pg/ml] | Stable phase | 23.2  (13.2, 43.5) | 13.1  (8.5, 20.1) | * |  | 10.3  (7.8, 31.3) | 8.6  (5.1, 13.0) | n.s. |  | n.s. |  | * |
|  | Exacerbation | 37.8  (29.5, 72.0) | 16.2  (11.1, 24.3) | *** |  | 38.4  (23.1, 86.0) | 14.9  (9.0, 20.2) | ** |  | n.s. |  | n.s. |
| **IL-8** [pg/ml] | Stable phase | 234.0  (146.2, 315.2) | 197.7  (127.0, 320.5) | n.s. |  | 322.7  (280.1, 345.3) | 114.9  (80.5, 233.5) | ** |  | n.s. |  | * |
|  | Exacerbation | 678.6  (393.2, 910.3) | 206.7  (137.3, 388.3) | * |  | 366.9  (220.3, 1534.7) | 204.0  (107.0, 311.0) | * |  | n.s. |  | n.s. |
| **NE** [pg/ml] | Stable phase | 311.9  (209.8, 483.8) | 177.3  (98.4, 500.6) | n.s. |  | 292.5  (237.8, 637.4) | 183.3  (99.0, 435.8) | n.s. |  | n.s. |  | n.s. |
|  | Exacerbation | 636.5  (241.8, 922.2) | 305.2  (138.8, 452.9) | * |  | 371.7  (283.3, 566.8) | 226.5  (185.6, 491.5) | n.s. |  | n.s. |  | n.s. |
| **TIMP-1** [pg/ml] | Stable phase | 4787.0  (3490.0, 7243.2) | 4899.5  (3738.4, 8766.5) | n.s. |  | 4499.6  (3289.9, 8138.9) | 6746.5  (4600.1, 9890.2) | n.s. |  | n.s. |  | n.s. |
|  | Exacerbation | 5669.0  (4490.4, 10427.0) | 5729.0  (3402.6, 8958.2) | n.s. |  | 6069.3  (4756.3, 9934.5) | 6096.7  (5426.5, 7982.5) | n.s. |  | n.s. |  | n.s. |
| **MMP-9** [pg/ml] | Stable phase | 3608.5  (3070.5, 4816.0) | 7236.5  (1493.1, 13389.7) | n.s. |  | 21402.0  (15000.4, 37195.1) | 5039.1  (1356.2, 7823.4) | ** |  | * |  | n.s. |
|  | Exacerbation | 47804.5  (7458.2, 192185.3) | 6372.0  (2412.6, 15161.3) | * |  | 31529.6  (9702.0, 64858.0) | 8198.1  (2784.0, 33040.0) | n.s. |  | n.s. |  | n.s. |
| HC: healthy controls; hRV: human rhinovirus; n.s.: not significant; * p < 0.05; ** p < 0.01; *** p < 0.001 | | | | | | | | | | | | |

**Table S9.** Inflammation markers in CF and healthy cohorts during stable phases and exacerbation in relation to hRV infection. Statistical differences were calculated using the Man-Whitney-U test. P-values <0.05 were considered statistically significant.
